# A single-cell atlas of the aging mouse ovary

**DOI:** 10.1038/s43587-023-00552-5

**Published:** 2024-01-10

**Authors:** José V. V. Isola, Sarah R. Ocañas, Chase R. Hubbart, Sunghwan Ko, Samim Ali Mondal, Jessica D. Hense, Hannah N. C. Carter, Augusto Schneider, Susan Kovats, José Alberola-Ila, Willard M. Freeman, Michael B. Stout

**Affiliations:** 1 Aging & Metabolism Research Program, Oklahoma Medical Research Foundation, Oklahoma City, OK USA; 2 Genes & Human Disease Research Program, Oklahoma Medical Research Foundation, Oklahoma City, OK USA; 3 Neuroscience Department, University of Oklahoma Health Sciences Center, Oklahoma City, OK USA; 4 Physiology Department, University of Oklahoma Health Sciences Center, Oklahoma City, OK USA; 5 Oklahoma City Veterans Affairs Medical Center, Oklahoma City, OK USA; 6 Nutrition College, Federal University of Pelotas, Pelotas, Brazil; 7 Arthritis & Clinical Immunology Research Program, Oklahoma Medical Research Foundation, Oklahoma City, OK USA

**Keywords:** Mechanisms of disease, Cellular signalling networks, Ageing

## Abstract

Ovarian aging leads to diminished fertility, dysregulated endocrine signaling and increased chronic disease burden. These effects begin to emerge long before follicular exhaustion. Female humans experience a sharp decline in fertility around 35 years of age, which corresponds to declines in oocyte quality. Despite a growing body of work, the field lacks a comprehensive cellular map of the transcriptomic changes in the aging mouse ovary to identify early drivers of ovarian decline. To fill this gap we performed single-cell RNA sequencing on ovarian tissue from young (3-month-old) and reproductively aged (9-month-old) mice. Our analysis revealed a doubling of immune cells in the aged ovary, with lymphocyte proportions increasing the most, which was confirmed by flow cytometry. We also found an age-related downregulation of collagenase pathways in stromal fibroblasts, which corresponds to rises in ovarian fibrosis. Follicular cells displayed stress-response, immunogenic and fibrotic signaling pathway inductions with aging. This report provides critical insights into mechanisms responsible for ovarian aging phenotypes. The data can be explored interactively via a Shiny-based web application.

## Abstract (web summary)

Isola, Ocañas et al. report age-related changes in the mouse ovarian transcriptome at single-cell resolution, demonstrating an increase in lymphocytes that corresponds to declines in collagen degradation and accumulation of multinucleated giant cells.

## Main

Ovarian aging has garnered substantial attention in recent years due to a large proportion of female humans choosing to delay childbearing^1^, which often causes difficulty with conception and carrying a pregnancy to full term^2^. As the ovary ages the local microenvironment changes in ways that reduce oocyte quality and increase the rate of follicular depletion, which eventually results in menopause. Menopause is associated with accelerated systemic aging^3^, greater chronic disease burden^4–6^ and increased all-cause mortality risk^7^. Therefore, a deeper understanding of the mechanisms that underlie ovarian aging is critically important to extending female fertility and attenuating age-related chronic disease onset.

It is well established that age-related ovarian follicular depletion is associated with increased mitochondrial dysfunction^8^, reactive oxygen species production^9,10^, inflammation^11–13^ and fibrosis^14,15^. However, very little is known about which cell types develop these phenotypes first and/or dominantly contribute to the changing local microenvironment. Moreover, it remains unclear whether cells in the follicle, stroma or both play mechanistic roles in the promotion of follicular depletion and ovarian failure. Recent work has sought to unravel the potential role played by ovarian stromal cells in ovarian health and disease^12,15,16^. We and others have reported that markers of cellular senescence and fibrogenesis increase within the ovarian stroma with aging^11,12,15^, although the specific cell types that become senescent and/or profibrotic remain unknown. In addition to increased senescence and fibrotic markers, the ovarian stroma also accumulates multinucleated giant cells (MNGCs) with advancing age, which may be related to the mechanisms that promote the aforementioned phenotypes^17^.

Due to the complex nature of ovarian function, which changes dynamically during aging, it has historically been challenging to elucidate the cell type-specific mechanisms that promote follicular depletion and ovarian failure. Data showing age-related changes in ovarian transcriptional programs within cellular subtypes are limited, particularly those pertaining to mice. Single-cell analyses of ovarian aging in nonhuman primates identified downregulation of antioxidant programs in aged oocytes and increased apoptosis in aged granulosa cells (GCs)^18^. In addition, single-cell analyses of human ovarian tissue are currently in progress^19^. However, mice represent the model organism most utilized for ovarian aging studies^20^ due to their short lifespan and ease of genetic manipulation for mechanistic studies. One spatially resolved analysis of mouse ovaries made strides in identifying age-related changes in ovarian cell populations^21^. However, this study used reproductively senescent (15-month-old) mice and lacked the resolution to identify specific cellular subtypes and important immune populations. To add to this growing body of work, we performed single-cell RNA sequencing (scRNA-seq) to identify age-related transcriptional changes in a cell type-specific manner within the mouse ovary at 3 and 9 months of age. We chose to analyze 3- and 9-month-old mice because they remain reproductively active at both ages, yet this age interlude represents a period when follicular density decreases markedly in conjunction with the emergence of age-related hallmarks^11^. This design and approach allowed us to determine how aging modulates cellularity and cellular phenotypes within the ovary before reproductive senescence, which is vital to the development of pharmacological approaches for extending the reproductive lifespan in female humans.

## Results

### scRNA-seq of the adult mouse ovary across reproductive ages

**Fig. 1:**
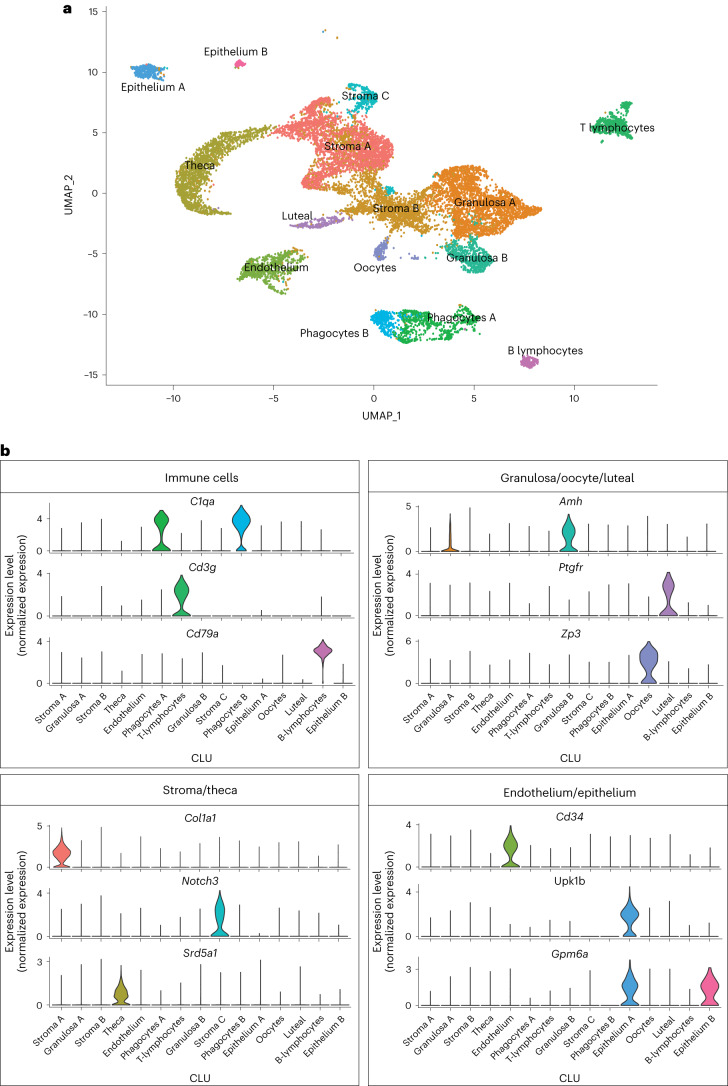
scRNA-seq of the mouse ovary. **a**,**b**, Whole-ovarian tissue was collected from 3- and 9-month-old C57BL/6 J mice and processed for 10X Genomics 3′ scRNA-seq. **a**, UMAP plot of age-combined ovarian cells. Clustering analysis revealed 15 distinct ovarian cell populations. **b**, Violin plots of specific marker genes for each ovarian cell type. scRNA-seq was performed in *n* = 4 ovaries per age group.

For evaluation of age-related changes in the ovary we collected ovaries from 3- and 9-month-old female mice (*n* = 4 per group) and performed scRNA-seq. The period from 3 to 9 months represents the time when the greatest decline in follicular reserve occurs and other hallmarks of aging begin to emerge^11^. Following quality control analyses, filtering and doublet removal, 14,504 cells remained for characterization. Cellular distributions of the number of genes detected, number of molecules and proportion of mitochondrial DNA before and after filtering are shown in Supplementary Fig. 1a. A mitochondrial DNA filtering parameter of 25% was used to ensure the inclusion of oocytes, because these are known to have more mitochondria than somatic cells^22,23^. In addition, mitochondrial-dependent apoptosis is involved in follicular atresia^24^ and thus a lower filtering threshold may result in loss of oocytes and follicular cells. Indeed, these clusters (CLUs) showed the highest mtDNA percentages among all identified CLUs (Supplementary Fig. 1b). Unbiased clustering and uniform manifold approximation and projection (UMAP) analysis revealed 15 distinct cellular CLUs (Fig. 1a). One CLU was present in only one sample and was subsequently identified as oviduct contamination (Supplementary Fig. 1c) and therefore these cells were removed from further analyses, which left 14,349 cells for downstream analyses. To assign cell type identity we used cell type-specific markers previously reported in the literature.

Collectively, the most common cell type was found to be stromal cells (*n* = 5,671), which segregated into three CLUs. One stromal CLU, referred to as Stroma A, was characterized by having a major *Col1a1* transcriptional signature^25^ as well as other stromal markers. A second CLU, Stroma B, was identified by the expression of several stromal markers (*Bgn*, *Ogn*, *Dcn*, *Lum*, *Col1a1* (ref. ^26^)). However, this CLU did not have any exclusive markers that were highly enriched. A third stromal CLU, Stroma C, was characterized by *Notch3* expression (Fig. 1b), which is classically viewed as a pericyte marker^26,27^. The second most common cell type was found to be GCs (*n* = 3,334), which segregated into two distinct CLUs, both displaying high expression of *Amh*^18,28^ (Fig. 1b). Other CLUs were identified as theca cells (TCs; *n* = 1,637; *Srd5a1* (ref. ^29^)), phagocytes (two distinct CLUs; *n* = 1,099; *C1qa* (ref. ^30^)), endothelial cells (*n* = 798; *Cd34* (ref. ^31^)), T lymphocytes (*n* = 728; *Cd3g* (ref. ^32^)), epithelial cells (two distinct CLUs; *n* = 450; *Upk1b* (ref. ^33^) or *Gpm6a* (ref. ^26^)), oocytes (*n* = 224; *Zp3* (ref. ^18^)), luteal cells (*n* = 206; *Ptgfr*^34^) and B lymphocytes (*n* = 202; *Cd79a* (ref. ^35^)) (Fig. 1b). Feature plots showing the specificity of these markers to each CLU can be found in Supplementary Fig. 1d. All markers for each CLU are listed in Supplementary Data 1.

**Fig. 2:**
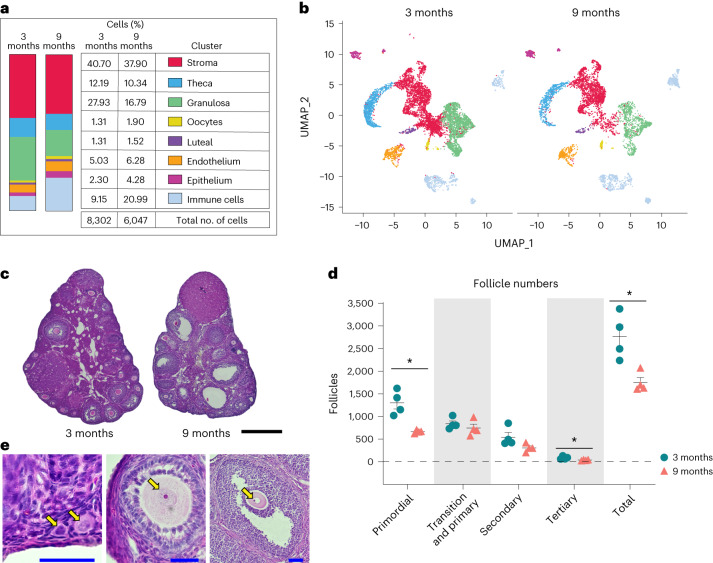
Age-related changes in ovarian cell populations. **a**, Numbers and percentages of cells in broad categories of cell type identity, by age. **b**, UMAP plot of ovarian cells, by age (color coding as in **a**). **c**, Representative images of H&E-stained ovaries. H&E staining and follicle counting were repeated for each biological replicate. Scale bar, 500 µm. **d**, Estimated numbers of follicles in 3- and 9-month-old ovaries. **e**, Representative images of follicles stained by H&E. Arrows (from left to right) indicate primordial, primary, secondary and tertiary follicles, respectively. Scale bars, 50 µm. Data presented as mean ± s.e.m. *FDR < 0.05, **FDR = 0.01, ***FDR = 0.005 by multiple two-tailed *t*-test with Benjamini, Krieger and Yekutieli correction for multiple comparisons. scRNA-seq was performed in *n* = 4 ovaries per age group. Exact *P* values shown in Source Data. Source data

Advancing age changed ovarian cellularity in our analyses (Fig. 2a,b and Supplementary Fig. 1e). The percentage of GCs was lower in 9-month-old ovaries, which can be explained by both declines in the number of primordial and tertiary follicles and a trending decline in secondary follicles observed in the aged mice (Fig. 2c–e). The most marked change in ovarian cellularity with advancing age was the greater than twofold increase in immune cells (Fig. 2a,b and Supplementary Fig. 1e).

In this study, although estrous cycle staging was not performed we used a bioinformatic approach to determine the estrous cycle stage of each mouse at the time of euthanasia. To do this we initially integrated our data with single-cell transcriptomic data from estrous cycle-staged mice^26^. That report showed major differences in single-cell transcriptional outcomes within GCs across estrous cycle stages, with unique features being observed during both proestrus and estrus. Following integration we then generated UMAP plots of GCs from mice in each estrous cycle stage and observed unique CLUs during both estrus and proestrus, which recapitulated the findings from Morris et al.^26^. We then created UMAP plots of GCs from each biological replicate in our study to determine whether they were in proestrus or estrus, which could potentially confound the interpretation of age-related transcriptional phenotypes (Supplementary Fig. 2a–d). We found that all four young mice, and three of the old mice, were in either diestrus or metestrus, which are transcriptionally indistinguishable^26^. One old mouse, Sample 5, was found to be in proestrus. We then used the list of transcripts, as reported by Morris et al.^26^ (Supplementary Data 1) to be upregulated during proestrus within GCs, to generate a module score for each sample, which enabled us to confirm that Sample 5 was in proestrus. These data are presented in violin plots in Supplementary Fig. 2e. We also performed principal component analysis of differentially expressed genes with age in our samples and observed that the only distinguishable clustering was that according to age (Supplementary Fig. 2f). Assessment of the potential impact of the proestrus sample on age-related phenotypic changes in GCs is discussed later in the manuscript following subcluster (SCL) analyses, although no major changes were observed.

For evaluation of intercellular signaling networks, CellChat analyses were performed. The most notable changes in cell signaling occurred among granulosa, stroma A and epithelium CLUs, with an overall increase in the number of interactions (Supplementary Fig. 3a) and interaction weights (Supplementary Fig. 3b) from 3 to 9 months of age. However, there was not a complete breakdown in cell-to-cell communication with aging.

### Ovarian immune cell changes with aging

**Fig. 3:**
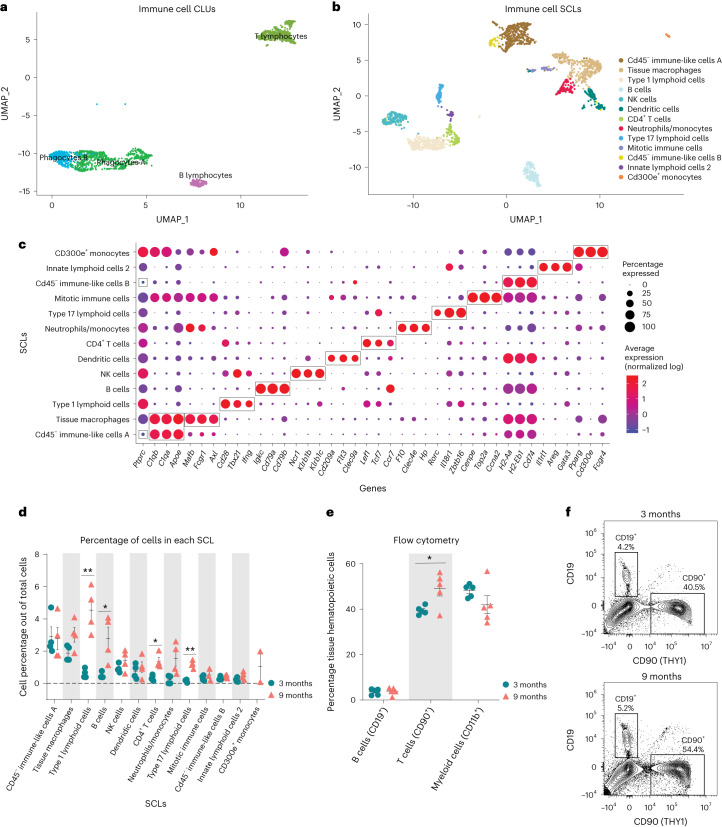
Immune cells accumulate in the ovary with age. **a**, UMAP of immune CLUs selected for subclustering analyses. **b**, UMAP of immune SCLs. **c**, Dot plot of markers used for SCL cell type identification. **d**, Percentages of cells in each immune SCL out of total cells. **e**, Percentages of immune cells out of tissue hematopoietic cells (following gating out of intravascular hematopoietic cells) by flow cytometry. **f**, Representative gating of CD19^+^ and CD90^+^ cells following gating out of tissue hematopoietic cells (intravascular CD45^−^, CD45^+^) in young and aged ovaries (see Supplementary Fig. 4a for complete gating strategy). For flow cytometry, six ovaries from mice in the same phase of estrous cycle were pooled (*n* = 5 per age group). scRNA-seq was performed in *n* = 4 ovaries per age group. Data presented as mean ± s.e.m. *FDR < 0.05, **FDR = 0.01, ***FDR = 0.005 by multiple two-tailed *t*-test with Benjamini, Krieger and Yekutieli correction for multiple comparisons. Exact *P* values given in Source Data. THY1, Thy-1 cell surface antigen. Source data

Following observation of the increased proportion of immune cells within the aged ovary, we performed subclustering of immune cells to determine which specific populations were changed with aging (Fig. 3a). Following reclustering, the original immune cell CLUs separated into 13 SCLs (Fig. 3b). Cell type identification for each SCL was performed based on known gene expression profiles and, with the help of databases Cellmarker2.0 (ref. ^36^) and ImmGen^37^, with the representative genes described in Fig. 3c. All markers for immune SCLs are listed in Supplementary Data 1. Because the overall immune cell proportion doubled from 3 to 9 months of age, percentages of total cells in each SCL were assessed and compared by age. Intriguingly, cell types showing the greatest increase with age were lymphoid, including B cells, conventional T cells and innate-like T cells. The biggest difference in abundance with age was in a SCL containing Type 1 lymphoid cells, as defined by the expression of *Tbx21* (ref. ^38^) and *Ifng*. These probably include CD8^+^ T cells, as well as other Type 1 innate-like T cells such as natural killer T cells (NKT cells), mucosal-associated invariant T cells (MAIT cells) and/or γδ T cells (γδT cells). Another SCL that significantly increased with age contained Type 17 lymphoid cells, as defined by the expression of *Rorc*^39^, which commonly represents innate lineage and expression of *Zbtb16* (refs. ^40,41^). Although transcriptional data cannot further discriminate within this SCL, it probably includes Type 17 NKT cells, MAIT cells, γδT cells and/or innate lymphoid cells Type 3 (ILC3), which was later confirmed by flow cytometry. Finally, the percentage of CD4^+^ T cells was also increased within the aged ovary (Fig. 3d). It should be noted that, due to single-cell transcriptomic limitations, other conventional T cells may also be contained within the CD4^+^ SCL. Conversely, neither natural killer (NK) cells nor ILC2s were significantly changed with age. Tissue macrophages and monocytes trended towards an increased proportion in the aged ovary but failed to reach statistical significance (Fig. 3d). One SCL, which was present only in the aged ovaries, was identified as monocytes that express CD300e, which negatively regulates T cell activation^42^. These CD300e^+^ monocytes may thus represent a compensatory mechanism to address T cell accumulation. However, this SCL might also represent blood monocyte contamination due to incomplete perfusion. Further investigation into this unique cellular population is warranted. Interestingly, although having an immune-like expression profile, two of the immune SCLs showed no expression of *Ptprc*, the gene that codes for CD45—the most well-established immune cell marker. These two SCLs were identified as CD45^−^ immune-like cells A and B.

**Fig. 4:**
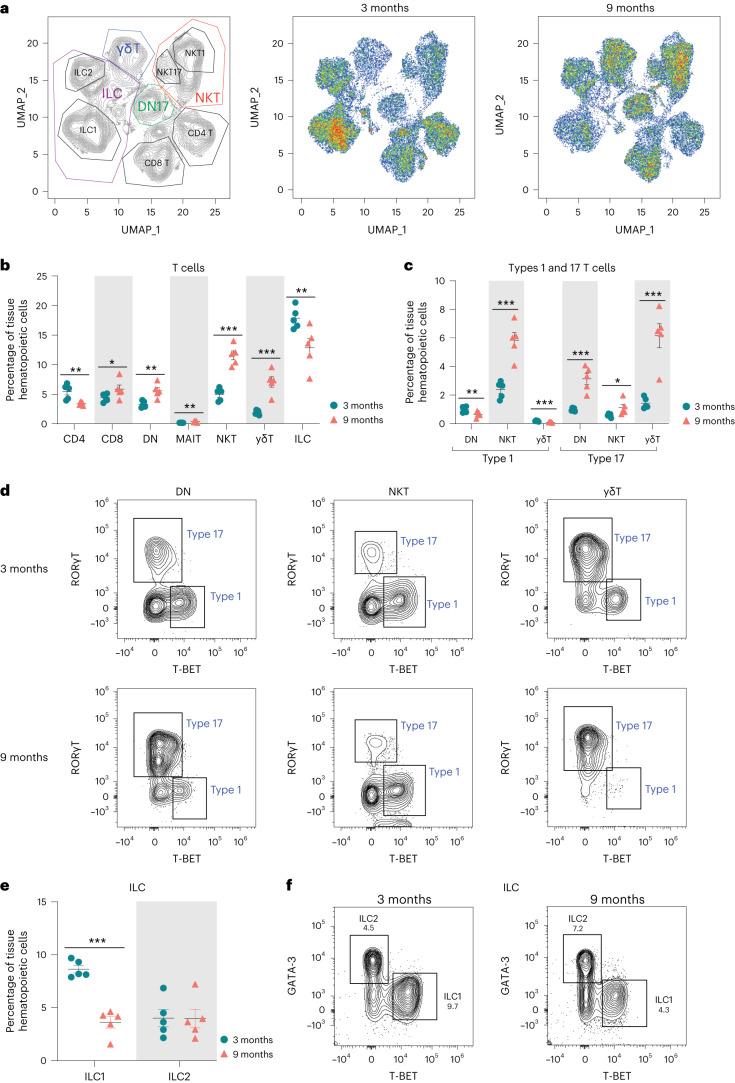
Lymphocyte populations are altered in the aged ovary. **a**, UMAP of flow cytometry panel of lymphoid cells, annotated using conventional gating strategies. The number of cells plotted was normalized to better observe differences in population distribution. **b**, Percentage of T cell subpopulations out of tissue hematopoietic cells. **c**, Percentage of Types 1 and 17 T cells out of tissue hematopoietic cells. **d**, Representative gating of Types 1 and 17 lymphoid cells in young and aged ovaries. **e**, Percentages of ICL1 and ILC2 out of tissue hematopoietic cells. **f**, Representative gating of innate lymphoid cells in young and aged ovaries. For flow cytometry, six ovaries from mice in the estrous cycle stage were pooled (*n* = 5 per age group). Data presented as mean ± s.e.m. *FDR < 0.05, **FDR = 0.01, ***FDR = 0.005 by multiple two-tailed *t*-test with Benjamini, Krieger and Yekutieli correction for multiple comparisons. Exact *P* values are given in Source Data. Source data

To confirm our transcriptional findings related to ovarian immune cell accumulation, we performed high-dimensional flow cytometry to identify age-related differences in the fraction of distinct immune cell types within the ovary. We first assessed the percentage and total number of broadly defined immune cell types, including B cells (CD19^+^), T cells and innate lymphoid cells (CD90^+^) and myeloid cells (CD11b^+^). We observed a higher percentage and number of CD90^+^ cells (T cells and innate lymphoid cells) in aged ovaries (Fig. 3e,f and Supplementary Fig. 4a), which aligns with the scRNA-seq findings. However, we did not observe changes in the percentage or number of myeloid cells or B cells (Fig. 3e,f and Supplementary Fig. 4a). Although the absence of change in myeloid cells was consistent with the scRNA-seq data, the B cells were incongruent. The proportion of B cells was found to increase with aging by scRNA-seq analyses, although this was not confirmed by flow cytometry. UMAP visualization of the flow cytometry data showed major shifts in immune cell subpopulations within the aging ovary (Fig. 4a). We then further characterized the CD90^+^ population and observed an increase in the percentage of CD8^+^ T cells, ‘double-negative αβ T cells’ (DNs), MAIT cells, NKT cells and γδTs and a decrease in CD4^+^ αβ T cells and innate lymphoid cells in aged ovaries (Fig. 4b and Supplementary Fig. 4b). To compare and contrast these findings with scRNA-seq clustering, we analyzed CD90^+^ cells based on expression of the effector-type-determining transcription factors for Type 17 lymphoid cells (RORγT) and Type 1 cells (T-BET) (Supplementary Fig. 4c). The increase in Type 1 lymphoid cells observed with scRNA-seq was confirmed by flow cytometry and further determined that the increase was driven by Type 1 NKT cells, while the proportions of Type 1 DN and γδT decreased with age (Fig. 4c,d). All RORγT subpopulations increased in proportion with age (DN, NKT, γδT) although the greatest magnitude of change occurred in Type 17 γδTs (Fig. 4c,d). Although the scRNA-seq data suggested the presence of ILC2—but not ILC1—subpopulations, the flow cytometry data identified both ILC1s (T-BET^+^) and ILC2s (GATA3^hi^). The flow cytometry data indicated a decrease in ILC1 percentages with advancing age (Fig. 4e,f). To determine the potential impact of estrous cycle stage on age-related changes in ovarian immune cell populations, we performed vaginal cytology on mice before euthanasia and ovarian flow cytometry analyses. We then plotted the mean fluorescence intensity of all markers assessed in each ovarian sample, in addition to identifying the estrous cycle stage of mice. We found that samples clustered by age, regardless of estrous cycle stage (Supplementary Fig. 4d), thereby indicating that estrous cycle stage is not a primary contributor to age-related changes in immune cell accumulation.

In addition to an overall increase in immune cells within the aged ovary, previous reports have also noted the accumulation of MNGCs as a hallmark of ovarian aging^17,43^. Although MNGCs were certainly removed from our single-cell suspensions during filtration, we were able to observe them in 9-month-old ovaries via histological assessment. Similar to previous reports^11,17,44^, we observed MNGCs in the aged ovary (Supplementary Fig. 5a), their presence corresponding to a rise in lipofuscin positivity (Supplementary Fig. 5b), which has been reported to be a marker of cellular senescence^45^. However, it should be noted that lipofuscin positivity strongly associates with increased numbers and size of MNGCs, which may not actually be cellular senescence as classically defined. Given the increased number of immune cells in the aged ovary that inherently express senescence-related genes (Supplementary Fig. 6a,b), it appears likely that ‘ovarian cellular senescence’ before estropause may represent increased immune cell accumulation. However, it should be noted that *Cdkn1a* and *Cdkn2d* expression increased in Type 17 lymphoid cells (Supplementary Fig. 6b–d) with advancing age, which could potentially represent a small subpopulation of cells that enter cellular senescence in the ovary.

### Subclustering of stroma and TCs reveals age-related changes

**Fig. 5:**
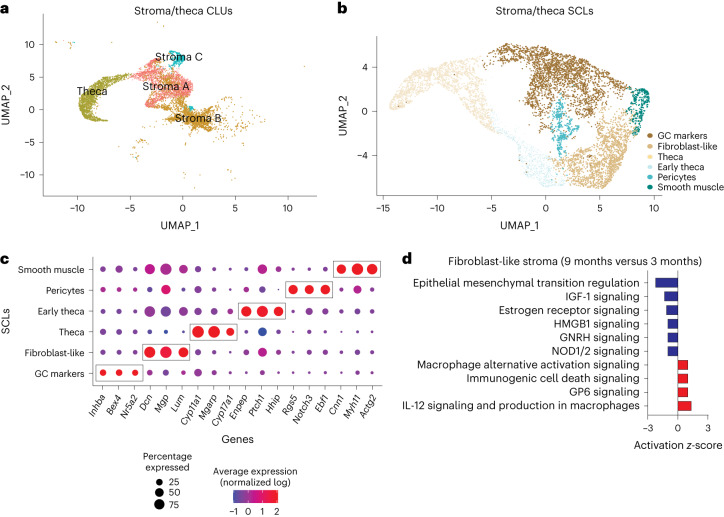
Subclustering of stroma and TCs. **a**, UMAP of stroma and TC CLUs used for subclustering. **b**, UMAP of stroma and TC SCLs. **c**, Dot plot of markers used for SCL cell type identification. **d**, IPA canonical pathways indicating activation or inhibition of specific pathways by aging in stromal fibroblast-like cells. scRNA-seq was performed in *n* = 4 ovaries per age group.

Several changes in the stroma have been observed with ovarian aging and are apparent before follicular exhaustion, including cellular senescent signatures^11^ and collagen deposition^12,14,15^. To identify potential cellular and molecular mediators of these phenotypes we performed subclustering of the stroma and TC CLUs (Fig. 5a,b). TCs were included with stroma because they dynamically differentiate from fibroblastic stromal cells during follicular maturation and thus share similarities with stromal cells^36^. This subclustering resulted in six distinct SCLs (Fig. 5b). Enriched markers in each SCL were used to infer cell type specificity (Fig. 5c). Notably, there was a SCL that expressed *Inhba*^37,38^, a marker of GCs, that was distinct and thus easily separated from other stroma and TC SCLs. The smooth muscle SCL was identified by the expression of *Myh11* (refs. ^46,47^), *Actg2* (ref. ^26^) and *Cnn1* (ref. ^48^). The pericyte SCL was enriched for *Notch3*, *Rgs5* and *Ebf1* (ref.^26^). The fibroblast-like SCL had enrichment of *Dcn*, *Mgp*^40^ and *Lum*^26^ whereas the TC SCL showed high expression of steroidogenic genes (*Cyp11a1*, *Cyp17a1* (ref. ^36^) and *Mgarp*^26^). The early TC SCL also showed high levels of steroidogenic genes including *Ptch1* and *Hhip*^41^, in addition to high expression of a fibroblast gene (*Enpp2* (ref. ^42^)), suggesting that these cells are in transition from stromal fibroblast-like cells to TCs. All markers for each stromal SCL are listed in Supplementary Data 1.

**Fig. 6:**
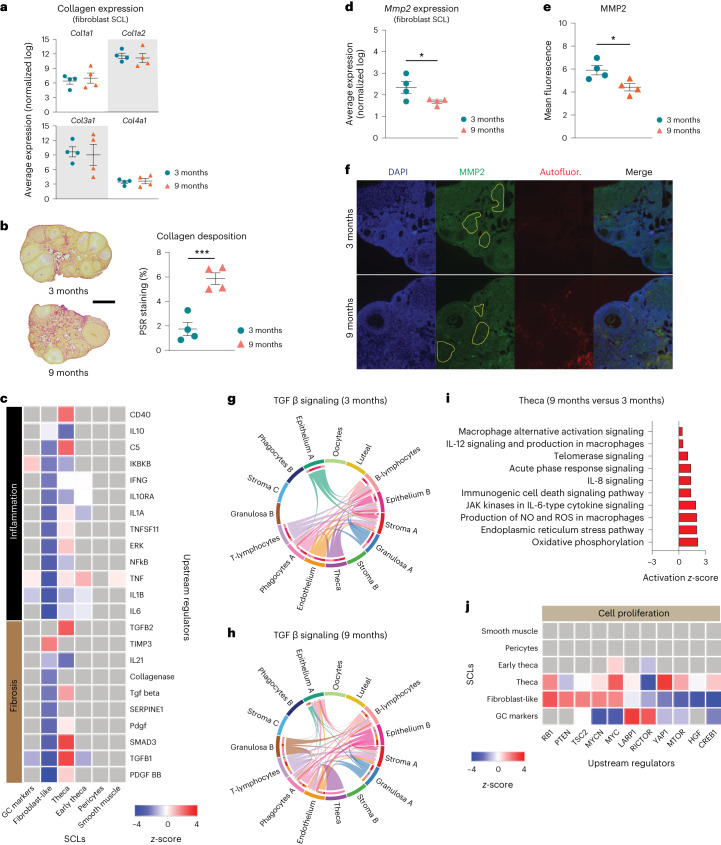
Biological significance of altered pathways in ovarian stroma and theca SCLs. **a**, Expression of collagen genes in the fibroblast-like stroma SCL does not change with age. **b**, Picrosirius red (PSR) staining of collagen deposition in 3- and 9-month-old ovaries. Scale bar, 500 µm. **c**, IPA upstream regulator analyses of age-related changes in stroma and TC SCLs (9 versus 3 months old, *z*-score) related to inflammation and fibrosis. **d**, Expression of *Mmp2* gene in fibroblast-like stroma decreases with age. **e**, MMP2 protein is decreased in aged ovarian stroma, as shown by immunofluorescence. **f**, Representative immunofluorescence images of MMP2 (green), DAPI (blue) and autofluorescence (red). Yellow-bordered areas represent stromal regions analyzed, avoiding follicles and autofluorescent regions. This assay was repeated independently for each biological replicate. **g**,**h**, CellChat chord diagrams of TGFβ signaling pathway interactions in 3-month (**g**) and 9-month ovarian CLUs (**h**). **i**, IPA canonical pathways indicating activation of specific pathways by aging in TCs. **j**, IPA upstream regulator analyses of age-related changes in stroma and TC SCLs (9 versus 3 months old, *z*-score) related to cell proliferation (*n* = 5 per age group). scRNA-seq was performed in *n* = 4 ovaries per age group. Data presented as mean ± s.e.m. *FDR< 0.05, **FDR = 0.01, ***FDR = 0.005 by one-tailed *t*-test (**a**,**b**) or two-tailed *t*-test (**d**,**e**). ROS, reactive oxygen species. Exact *P* values shown in Source Data. Source data

Following SCL identification, a list of genes differentially expressed by the different ages in each SCL was imported into the software Ingenuity Pathway Analysis (IPA) to infer biological function. Fibroblast-like stromal cells showed an age-related upregulation of pathways related to tissue remodeling^49,50^ (Fig. 5d). Tissue remodeling occurs following ovulation and in cases of follicular and luteal atresia^16^. To our surprise, collagen expression was unchanged by aging in ovarian fibroblast-like SCL (Fig. 6a) despite greater collagen deposition by 9 months of age (Fig. 6b). Interestingly, the collagenase pathway was downregulated in the fibroblast-like SCL (Fig. 6c). Further analyses revealed that the expression of matrix metalloproteinase 2 (*Mmp2*), a key enzyme involved in collagen degradation^51^, was decreased in the fibroblast-like SCL with advancing age (Fig. 6d). We also observed an age-related decrease in MMP2 protein in the ovarian stroma by immunofluorescence (Fig. 6e,f). These findings suggest that ovarian collagen accumulation is at least partially mediated by a reduction in collagen degradation, which supports our transcriptional findings. Fibroblast-like cells also showed downregulation in hormonal signaling (estrogen receptor and gonadotropin-releasing hormone; Fig. 5d), which is somewhat surprising given that hormone levels, estrous cyclicity and fertility are generally stable at 9 months of age^52^. These results indicate that changes in the local microenvironment may contribute to endocrine dysfunction in the ovarian stroma independently of changes within the follicle. However, fibroblast-like cells showed a downregulation in upstream regulators associated with inflammation and fibrosis (Fig. 6c), which could be a compensatory response to increases in proinflammatory and profibrotic signaling from other cell types, as well as immune cell accumulation. In other tissues, fibroblasts are known to play immunosuppressive roles in the regulation of chronic inflammation^53^. In contrast to fibroblast-like cells, TCs showed a significant upregulation in several upstream regulators of fibrogenesis including TGFB1, TGFB2 and SMAD3 (Fig. 6c), which suggests that TCs may be one of the earliest cell types involved in generation of the signaling cascade for collagen production and deposition.

In contrast to fibroblast-like cells, TCs showed significant upregulation in several upstream regulators of fibrogenesis including TGFB1, TGFB2 and SMAD3 (Fig. 6c), which suggests that these may be one of the earliest cell types involved in generating the signaling cascade for collagen production and deposition. When looking at TGFβ intercellular communication among the original CLUs, we note that TCs signal to several cell types in the young ovary (Fig. 6g), which was expected, due to its important role in intrafollicular cell-to-cell communication^54^. In contrast, TGFβ signaling in TCs from aged ovaries became exclusive to immune cells (Fig. 6h). TGFβ signaling has previously been associated with the regulation of chemotaxis, activation and survival of lymphocytes^55,56^, which may explain our finding of increased lymphocyte accumulation in the aged ovary. Additionally, the Granulosa B CLU showed only TGFβ signaling in the old ovary (Fig. 6g,h). These data suggest that interactions between ovarian follicular cells and immune cells are probably important for perpetuation of fibrotic signaling with aging. TCs also showed a modest age-related upregulation in inflammation and cell stress-response pathways (Fig. 6i), which were mirrored by upstream regulators involved in cellular proliferation including MTOR, YAP1 and RB1 (Fig. 6j).

### Aging affects granulosa, oocyte and luteal cell SCLs

**Fig. 7:**
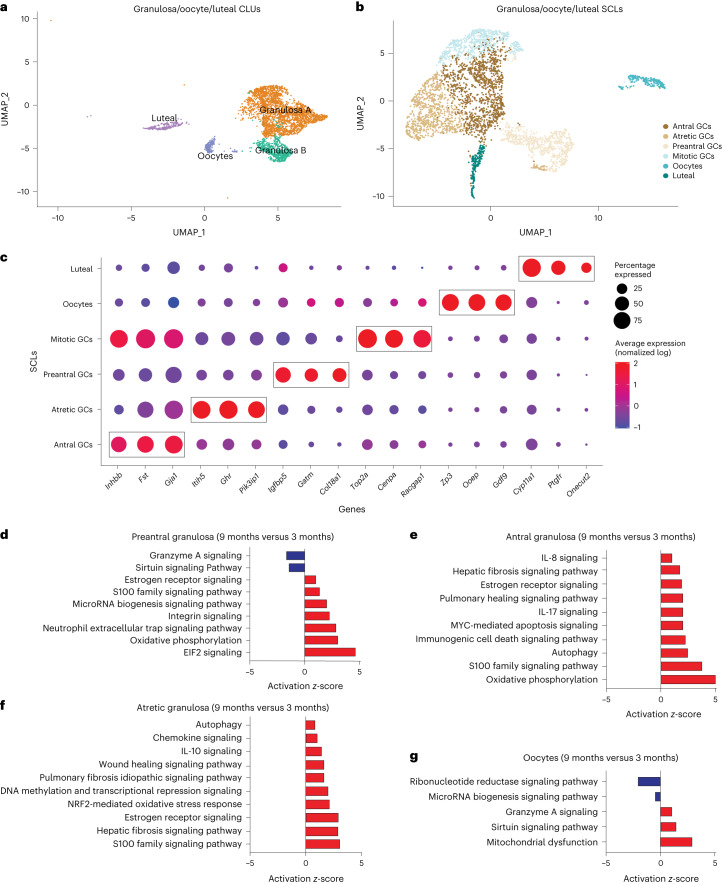
Subclustering of GCs, oocytes and luteal cells. **a**, UMAP of granulosa, oocyte and luteal cell CLUs used for subclustering. **b**, UMAP of granulosa, oocyte and luteal SCLs. **c**, Dot plot of markers used for SCL cell type identification. **d**–**g**, *Z*-scores indicating activation or inhibition of pathways during aging by IPA analysis in preantral GCs (**d**), antral GCs (**e**), atretic GCs (**f**) and oocytes (**g**). scRNA-seq was performed in *n* = 4 ovaries per age group.

Cellular populations of GCs, oocytes and luteal cells were subclustered into six distinct SCLs (Fig. 7a–c). GCs were further segregated into four distinct SCLs that were identified as being part of follicles at different stages of development. These SCLs included preantral, antral, mitotic and atretic GCs. GCs signal to oocytes to provide cues related to the local ovarian microenvironment, in addition to conversion of androgens to estrogens, which are released into the systemic circulation for feedback signaling in the brain^57^. GCs from immature follicles that have not yet developed an antrum are referred to as preantral GCs and were identified by enrichment in *Igfbp5* (ref. ^58^) and *Gatm*^59^ expression. GCs from antral follicles (antral GCs) were identified by enrichment of *Inhbb*^60,61^. A separate GC SCL, which we refer to as mitotic GCs, expressed *Inhbb* similarly to antral GCs but showed enriched expression of cell division markers *Top2a* (ref. ^62^) and *Racgap1* (ref. ^63^). Last, GCs from follicles undergoing atresia were found to be enriched for *Pik31p1* (refs. ^26,64^), *Itih5* (ref. ^26^) and *Ghr*^26^. Oocytes were readily identified by the expression of classical markers *Gdf9*, *Ooep* and *Zp3* (ref. ^18^). Luteal cells, which consist of remnant GCs and TCs following ovulation and constitute the corpus luteum^65^, were identified by the expression of *Ptgfr*^34^. All markers for each GC SCL can be found in Supplementary Data 1.

As alluded to above, differentially expressed genes from each SCL were examined with IPA software to infer biological function. Interestingly, preantral, antral and atretic GCs all displayed an age-related increase in pathways related to proinflammatory stress and fibrogenesis (Fig. 7d–f). These observations corresponded to a significant increase in the mitochondrial dysfunction pathway within oocytes (Fig. 7g). Upstream regulators related to proinflammatory stress that were found to be increased in GC SCL included IFNG, TNF, IL15 and IL1A (Supplementary Fig. 7a). Additionally, upstream regulators related to fibrogenesis were found to be increased in GC SCL including TGFB1, TGF-beta and SMAD3 (Supplementary Fig. 7a). As expected, the atretic GC SCL was enriched for changes in proinflammatory stress and fibrosis pathways, with 9-month-old mice having greater activation of these pathways than 3-month-old mice. Therefore, it appears that aging exacerbates inflammatory and fibrotic responses that are required for ovarian remodeling following ovulation and follicular atresia.

We also examined intercellular C–C motif chemokine ligand (CCL) signaling due to its role in immune cell migration and chemotaxis. We found that, by 9 months of age, granulosa CLUs expressed CCL signaling molecules, which was not observed in 3-month-old mice (Supplementary Fig. 7b,c). At 9 months of age GC CCL signaling was exclusive to the phagocyte A CLU. This suggests that inflammatory signaling from the follicle may contribute to alterations in immune cell phenotypes as a function of age. Interestingly, there was a breakdown in the anti-Mϋllerian hormone signaling pathway between oocytes and other follicular cells by 9 months of age (Supplementary Fig. 7d,e). The anti-Mϋllerian hormone pathway is critical for regulation of follicular maturation^66^ and is commonly used as a marker of follicular reserve^67^. This breakdown in signaling therefore signifies early changes that may impact reproductive outcomes.

Because GC transcriptional changes have been reported during proestrus, coupled with one of the 9-month-old mice (Sample 5) analyzed herein being in proestrus, we performed additional analyses to ensure that estrous cycle stage did not confound our age-related interpretations. For this analysis we removed those genes that were found to be differentially expressed in both proestrus^26^ and aging in granulosa SCLs, and reran the pathway analyses. The removal of these genes did not affect age-related changes in GCs. As a secondary confirmation we then completely removed Sample 5, reran the pathway analysis and found that age-related outcomes were unchanged (Supplementary Fig. 7f). These observations provide additional evidence that our interpretations related to the mechanisms promoting ovarian aging are unaffected by estrous cycle stage.

### Endothelial and epithelial SCLs are mildly affected by age

**Fig. 8:**
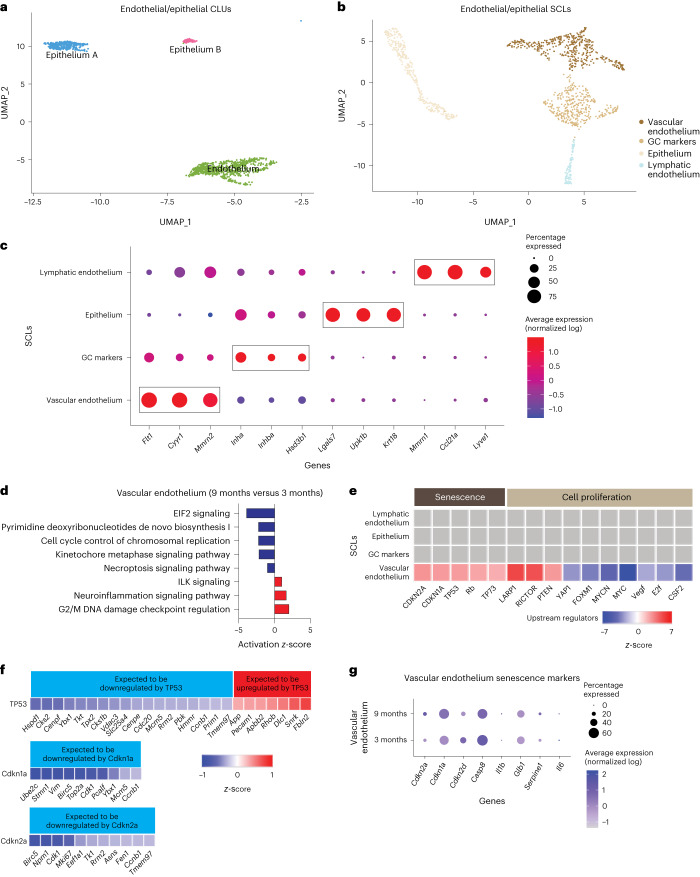
Subclustering of endothelial and epithelial cells. **a**, UMAP of endothelial and epithelial CLUs used for subclustering. **b**, UMAP of endothelial and epithelial SCLs. **c**, Dot plot of markers used for SCL cell type identification. **d**, *Z*-scores indicating activation or inhibition of pathways altered with aging, as evidenced by IPA analysis, in vascular endothelial cells. **e**, *Z*-scores indicating activation or inhibition of upstream regulators during aging, as evidenced by IPA analysis, in endothelial and epithelial SCLs. **f**, *Z*-scores indicating up- or downregulation of genes by genes *TP35*, *Cdkn1a* and *Cdkn2a*. **g**, Dot plot showing expression of cell senescence markers in vascular endothelial cells in 3- and 9-month-old ovaries. scRNA-seq was performed in *n* = 4 ovaries per age group.

Endothelial cells are essential components of blood and lymphatic vessels. The ovary is a highly vascularized organ with blood vessels that travel through connective tissue to assist with hormone/nutrient trafficking and waste removal^68^. The ovary also has a rich lymphatic network, closely associated with the blood vasculature, that is involved in immune cell trafficking^69^. Epithelial cells comprise the ovarian surface, facilitate repair following ovulation and dynamically expand and contract during cyclic ovarian changes^70^. In addition, epithelial cells share a common progenitor with GCs^71^. Endothelial and epithelial CLUs were subclustered (Fig. 8a), resulting in four distinct SCLs (Fig. 8b). Vascular and lymphatic endothelia were separated and identified by the enrichment of specific cellular markers (Fig. 8c). Vascular endothelial cells showed enrichment of *Flt1* (ref. ^72^) and *Mmrn2* (ref. ^73^) whereas lymphatic endothelial cells were enriched for *Lyve1* (ref. ^74^) expression. All markers for each endothelial/epithelial SCL are listed in Supplementary Data 1.

Similar to findings in stroma/theca SCLs, one of the endothelial/epithelial SCLs was found to be enriched for GC markers and was removed from further analyses; no age differences were observed in this GC SCL. The only epithelial/endothelial SCL that showed age-related changes in the pathway analyses was the vascular endothelium; this SCL was found to have modestly increased DNA damage regulation (Fig. 8d). Another interesting observation was that upstream regulators of cellular senescence (TP53, CDKN1A and CDKN2A) were found to be increased with aging in vascular endothelial cells (Fig. 8e). Further analysis of genes downstream of TP53, CDKN1A and CDKN2A found these to be altered in directionality, consistent with the activation of these upstream regulators (Fig. 8f). This is consistent with studies indicating that vascular endothelial cells are highly susceptible to becoming senescent^75^. In the ovary, cyclicity requires constant vascular remodeling^68^, which we speculate may promote senescence in endothelial cells. However, despite observing increased TP53, CDKN1A and CDKN2A upstream regulator activity in the vascular endothelium SCL, the expression of *Cdkn1a* and *Cdkn2a* was unaffected by aging (Fig. 8g) whereas *Tp53* was not detected in our scRNA-seq analysis. Therefore, it remains unclear whether the vascular endothelium is a source of senescent cells in the aged ovary.

## Discussion

In this report we assessed age-related changes in the mouse ovarian transcriptome at single-cell resolution. During aging, ovarian follicular reserve declines and there is a concomitant deterioration of the ovarian microenvironment as evidenced by increased inflammation and fibrosis. Importantly, these changes occur before follicular exhaustion^11^ and probably contribute to decreased oocyte quality and diminished reproductive success until ovarian insufficiency occurs. However, specific cellular contributions to ovarian aging phenotypes are not yet elucidated, limiting the development of interventional approaches to extend female fertility. Whole-ovarian bulk transcriptomic assessments can provide results that are difficult to interpret due to potential changes in cell heterogeneity that occur during aging. Cell sorting can overcome this limitation but relies on specific antibodies or Cre-reporter systems to target specific cell types. scRNA-seq, on the other hand, is a useful tool for simultaneous measurement of transcriptomic changes in all ovarian cell types during the aging process. Previous ovarian scRNA-seq analyses have identified the molecular signatures of specific cell types and changes in individual cell populations^18,26,28,59,76,77^. In mice, estrous cycle^26^ and primordial follicle assembly^76^ result in dynamic changes in specific ovarian cellular populations. However, the specific cellular changes occurring during ovarian aging are still being elucidated, especially with regard to the critical period of diminished fertility that occurs long before follicular exhaustion. Because mice are the primary model organisms used for ovarian aging experiments^20^, the single-cell ovarian aging atlas presented herein serves as a crucial resource for the field. In nonhuman primates, scRNA-seq has provided mechanistic insights into changes associated with ovarian aging^18^. However, this study was conducted with animals in the perimenopausal state^78^ when alterations in cyclicity and hormone levels are perturbed. Our primary goal was to identify early changes that occur in reproductively aged mice before the periestropausal period. At 9 months of age mice experience declined ovarian reserve^11^ but remain fertile^79^, modeling a critical age when female humans seek to have children and experience difficulty conceiving^2^. In humans a marked increase in embryonic aneuploidy is observed in embryos from donors, starting around 35 years of age^80^, suggesting a loss in oocyte quality. With our approach, we differentiated all of the main cell types expected in the ovary and further subdivided them to determine specific cellular populations altered by the aging process.

Our scRNA-seq results show that the proportion of immune cells in the ovary doubles between 3 and 9 months of age. The most marked increase in immune cells within aged ovaries occurred in Types 1 and 17 lymphoid cells. A recent scRNA-seq assessment of ovarian immune cells reported an increase in CD4^−^CD8^−^ T cells, deemed double-negative T cells, in aged ovaries^81^. Many of the Types 1 and 17 lymphoid cells described in the present study, including αβ DNs, Type 17 NKTs, MAITs, γδTs and a substantial proportion of Type 1 NKTs, are CD4^−^CD8^−^ and would therefore be included within the broad double-negative T cell population described in the previous study. Consistent with the previous report, our data indicate that many of these populations increase with age. However, our data provide better granularity and revealed changing in the aging ovary of specific populations of Types 1 and 17 lymphoid cells that had not previously been evaluated.

Specifically, we determined that the immune subpopulations that most consistently increased in the aged ovary were Type 1 NKTs and Type 17 γδTs, both of which are innate T cells. Our data thus raise interesting questions about the functional roles of innate T lymphocytes in both the young and old ovary. Innate T cells are tissue-resident cells thought to contribute to the maintenance of tissue homeostasis and serve as sentinel cells in the detection of infectious agents, neoplastic cells or aberrant cellular damage^82^. In the female reproductive tract, innate lymphocytes show high functional diversity with both beneficial and detrimental effects^83^. However, the role of innate lymphocytes in the ovary is an unexplored subject. Type 17 γδTs play critical roles during fibrosis in a variety of organs, including liver^84,85^, kidney^86^, lung^87^ and heart^88^. However, whether these cells are protective or deleterious in fibrosis appears to be dependent on both tissue and context^89,90^. For instance, age-related accumulation of Type 17 γδTs contributes to chronic inflammation in adipose tissue^91^. In the ovary, expansion of Type 17 γδTs could either promote fibrosis or be a compensatory attempt to dampen the fibrotic environment. Little is known about the role of Type 1 NKTs in the ovary. In general, NKTs provide important immune responses during injury, repair, inflammation and fibrosis in the liver and lung^87^. Further mechanistic studies assessing the roles of these cells in ovarian fibrosis and inflammation are warranted.

Under normal conditions, tissue-resident lymphocytes are maintained through slow homeostatic proliferation^92^, as opposed to adaptive T cells that are generally recruited from the circulation^93^. The mechanisms underlying increased innate lymphocyte numbers in the aged ovary may be a result of increased proliferation in response to tissue remodeling and/or local inflammatory cytokine production. This hypothesis is consistent with increased IFNG, TNF, IL15 and IL1A upstream regulator activity in GC SCLs. Alternatively, circulating lymphocytes could be recruited into tissue in response to similar proinflammatory signals^94^. Chronic ovarian inflammation caused by autoimmune disease also promotes lymphocyte accumulation^95^, and female humans with autoimmune diseases often experience ovarian insufficiency^96^. This suggests an association between the accumulation of lymphocytes and age-related follicular declines, which merits future interrogation.

Although we detected an increase in the proportion of B cells within the scRNA-seq data at 9 months of age, this was not confirmed by flow cytometry. A previous report showed that the proportion of ovarian B cells slightly increased from 2 to 6 months of age but then returned to basal levels by 12 months of age^13^. This suggests that B cell numbers are highly variable in the aging ovary. Within our scRNA-seq data we also detected an increase in CD4^+^ T cells in the 9-month-old ovary. Although the proportion of CD4^+^ T cells decreased between 3 and 9 months of age according to flow cytometry, the absolute number of these cells increased with age. The accumulation of CD4^+^ T cells was previously reported in the aged ovary^13^, although these changes were not observed until 12 months of age. This suggests that the accumulation of CD4^+^ T cells may begin to occur at 9 months of age.

The literature addressing age-related changes in ovarian myeloid cells is conflicting^13,81,97^, which may be due to differences in the methods employed and ages evaluated. We found no significant changes in ovarian myeloid cell accumulation by 9 months of age through scRNA-seq or flow cytometry analyses. At this juncture it remains unclear whether myeloid cells actually increase in the ovary with advancing age, but the fusion of monocytes and macrophages into MNGCs^98^ could confound these interpretations. As addressed previously, MNGC accumulation is a hallmark of the aged ovary^49^. Previous reports suggest that MNGCs may contribute to the age-related deterioration of the ovarian microenvironment^17^. However, the mechanisms that promote MNGC formation and their physiological ramifications in the ovary remain unresolved. The best-characterized MNGCs are osteoclasts, which are implicated in bone remodeling through phagocytosis of apoptotic cells and collagen fragments^99^. In the ovary, MNGCs may also serve similar functions because phagocytic processes control tissue remodeling during follicular atresia, corpora lutea regression and ovulation. Interestingly, because estrogen induces osteoclast apoptosis, menopausal-related loss of estrogen action accelerates bone loss in humans^100^. Similarly, decreased estrogen signaling in the aged ovary may promote MNGC accumulation. Lymphocytes, which are increased in the aged ovary, also contribute to MNGC formation through cytokine-mediated mechanisms^101^. For example, Il17a (the primary cytokine produced by Type 17 lymphoid cells) is associated with osteoclast formation^102^, which suggests that Type 17 lymphoid cells may contribute to MNGC formation in the aged ovary. Similarly, IFNG is known to induce MNGC formation^103,104^. Our data show that the primary source of *Ifng* production in the ovary is Type 1 lymphoid cells, which accumulate in the ovary during aging. Additional studies will be required to further elucidate the mechanisms underlying MNGC formation in the ovary and the role they play in ovarian function and aging processes.

The aforementioned accumulation of lymphoid cells and MNGCs almost certainly contributes to pathological changes that occur in the aged ovary. We speculate that they crosstalk with follicular cells to induce immunogenic responses that adversely affect the local microenvironment. For instance, GCs show upregulated IL-17 and IFNG pathway activity with advancing age, although these genes are dominantly expressed in lymphocytes. This supports the notion that lymphoid cells produce proinflammatory ligands that alter GC transcriptional networks. We also found that GCs upregulate oxidative stress pathways, which we surmise may occur in response to the proinflammatory environment. This finding is supported by a scRNA-seq analysis of aged nonhuman primate ovaries that also show declines in oxidoreductase pathway activity in GCs^18^. These similarities were observed despite the nonhuman primate ovaries being collected during the perimenopausal period, whereas the mouse ovaries were collected before the periestropausal period. In addition to immunogenic responses, we also found that GCs and TCs display age-related induction of fibrotic responses as evidenced by increased TGFβ pathway activation. In alignment with previous observations^12,14,15^, we saw increased ovarian collagen deposition by 9 months of age. Because collagen is primarily produced by stromal fibroblast-like cells^14,105^, we evaluated the expression of collagen genes in this SCL but found no age-related changes. Conversely, the collagenase pathway and MMP2 expression were downregulated in the stroma of aged ovaries, suggesting that collagen accumulation occurs due to declines in degradation. It is also noteworthy that TCs upregulate cell proliferation pathways (for example, mTOR), which increase with aging^106^ but, more importantly, have been linked to the activation of primordial follicles^107^.

Although the data presented herein serve as an important tool for hypothesis generation, there are a few limitations that should be noted. Cell preparation for scRNA-seq and flow cytometry requires filtration steps that remove MNGCs and large oocytes from analyses. Future studies employing laser-capture microscopy and single-nuclei sequencing would allow for interrogation of these large-cellular populations. Even though the mouse is the most common model organism used for ovarian assessment, there are important aspects of their ovarian biology that differ from nonhuman primates and humans: for example, humans are a mono-ovulating species whereas mice are polyovulating and therefore follicular selection signaling probably differs between these species. In addition, virgin mice were used in this study, which does not account for the impact of pregnancy on ovarian aging dynamics. The examination of additional time points following reproductive senescence should also be considered, because it would provide critical insights related to how the ovary changes following the loss of fertility, which may impact systemic aging mechanisms. Finally, estropause in mice does not completely recapitulate the changes observed in nonhuman primate and human menopause. As such, other models of ovarian aging should be evaluated before definitive conclusions are drawn.

In summary, this report provides insight into changes that occur within the aging murine ovary at single-cell resolution. We report that, by 9 months of age, when mice remain fertile and reproductively active, immune cell accumulation is already doubled with the greatest changes being observed in innate lymphocytes including Type 1 NKTs and Type 17 γδTs. We also demonstrate that GCs and TCs upregulate stress-response, immunogenic and fibrotic signaling pathways with aging. These changes correspond to declines in collagenase expression in the stroma, which we surmise contributes to collagen accumulation with age. These changes were accompanied by accumulation of MNGCs with advancing age, which accounted for the vast majority of lipofuscin positivity in aged ovaries. This observation, coupled with the increase in immune cells that commonly express transcriptional markers of cellular senescence, probably contributes to the previously reported increase in ovarian cellular senescence with advancing age. Collectively, our findings provide insights into the underlying mechanisms that promote chronic ovarian inflammation and fibrosis with advancing age and serve as an important resource for the field.

## Methods

### Animals and tissue collection and dissociation for scRNA-seq

All animal procedures were approved by the Institutional Animal Care and Use Committee at the Oklahoma Medical Research Foundation. C57BL/6 J (strain no. 000664) female mice (*n* = 8) were purchased from the Jackson Laboratory and acclimated to the Oklahoma Medical Research Foundation animal facility before ovary collection. During acclimation, mice were maintained at 22 ± 0.5 °C on a 12/12-h light/dark cycle and had ad libitum access to food (LabDiet no. 5053, Purina) and water. At 3 (*n* = 4) or 9 months of age (*n* = 4), mice were anesthetized with isoflurane and euthanized by exsanguination via cardiac puncture. Perfusion was performed with 1× PBS, and ovaries collected and dissected. One ovary from each mouse was dissociated using Multi Tissue Dissociation Kit 1 (cat. no. 130110201, Miltenyi Biotec), following the manufacturer’s instructions, to create a single-cell suspension in Dulbecco’s PBS (cat. no. 14287080, Gibco).

### scRNA-seq library construction

scRNA-seq libraries were constructed with the Chromium Single Cell 3ʹ GEM, Library & Gel Bead Kit v.3 (cat. no. 10000075, 10X Genomics), according to the manufacturer’s instructions, as briefly described below. Following the creation of ovarian single-cell suspensions, cells were counted on a MACSQuant10 flow cytometer and diluted to 1,000 cells µl^−1^ to target the recovery of 5,000 cells per sample during scRNA-seq encapsulation. The diluted cells, master mix, gel beads and partitioning oil were added to the Chromium Single Cell B Chip (cat. no. 10000073, 10X Genomics) and loaded into a Chromium controller (cat. no. 1000204, 10X Genomics) to generate the gel beads-in-emulsion (GEMs) for downstream library preparation. GEMs were then transferred to PCR strip tubes and incubated in a thermocycler to perform reverse transcription in GEMs. Following reverse transcription in GEMs, the recovery agent was aspirated and complementary DNA was cleaned using the Dynabeads MyOne SILANE reagent included in the scRNA-seq kit. The cDNA was amplified and then cleaned using SPRISelect reagent beads (cat. no. B23318, Beckman Coulter). The cDNA was quality checked using a High Sensitivity D5000 ScreenTape (cat. no. 5067-5592, Agilent) run on a TapeStation 2200 (cat. no. G2964AA, Agilent). An aliquot of 25% of amplified cDNA was carried forward to library preparation. Libraries were quantified by quantitative PCR and quality checked on a High Sensitivity D1000 ScreenTape (cat. no. 5067-5584, Agilent) on the TapeStation 2220. Libraries were normalized, pooled and sequenced on a NovaSeq6000 PE150 flow cell. The sequence depth obtained was ~50,000 reads per cell.

### scRNA-seq quality control and data analysis

Fastq files were generated and demultiplexed from raw base call (bcl) files using the cellranger mkfastq pipeline. Alignment, filtering, barcode counting and unique molecular identifier counting were conducted with the cellranger count pipeline using the refdata-gex-mm10-2020-A reference transcriptome with default settings. The resultant out files were loaded into R Studio (v.4.2.2) using the ‘load10X’ function of the SoupX package^108^. The SoupX pipeline was then used to estimate and remove ambient RNA contamination before conversion to Seurat objects^109^. Samples were then merged to create a single Seurat object and filtered based on the number of features (>200) and percentage mitochondrial transcripts (<25%). Genes expressed in fewer than three cells were removed from analysis. Genes representing ribosomal contamination (*Malat1*, *Gm42418*, *Gm26917*, *Ay036118*)^110,111^ were causing technical background noises and were thus removed from the analysis to improve subclustering. Variable features were identified in Seurat before scaling data and running principal component analysis. The JackStraw method was used to determine the dimensionality of the dataset, and UMAP analysis was generated in Seurat. Doublets were identified and removed using the DoubletFinder package^112^, with 5% doublets expected. Other parameters (pN=0.25, pK=0.01) were generated using DoubletFinder sweep statistics. Samples were coprojected on a UMAP that was used to determine that there were no batch effects and that further data integration was not necessary. Seurat was used to find differentially expressed genes by CLU and age and to generate plots presented in the figures (that is, DimPlot, VlnPlot and DotPlot). Module scores were calculated by the average expression levels of each program at single-cell level, subtracted by the aggregated expression of control feature sets. To assess cell-to-cell interactions between different ovarian cell types in young and aged mouse ovaries we used CellChat45 (v.1.6.1), which is based on the expression of known ligand–receptor pairs^113^.Gene lists were imported into IPA 01.12 (Qiagen Bioinformatics) software to assess pathway/biological function enrichment analysis.

### Histology

The remaining ovary from each mouse was collected into 4% paraformaldehyde, processed and serially sectioned. To determine ovarian reserve, one of every six serial sections from the whole ovary was hematoxylin and eosin (H&E) stained and follicles from each state were counted as described previously^114^. The number of preantral follicles (primordial, primary and secondary) was multiplied by six to account for sections that were not evaluated and then multiplied by two to account for both ovaries. Due to their large size, antral follicles were multiplied by three to account for sections that were not evaluated and then multiplied by two to account for both ovaries. Picrosirius red staining for collagen deposition and Sudan black staining for lipofuscin accumulation were performed on one randomly assigned section from the midline of each ovary and analyzed as previously described^11,12^.

### Immunofluorescence

Evaluation of MMP2 protein in the ovarian stroma was performed in paraffin-embedded ovarian sections as previously described^115^. Slides were incubated with primary rabbit anti-MMP2 antibody (1:100; cat. no. 87809, BioLegend) overnight, followed by secondary goat anti-rabbit IgG Alexa Fluor 488 antibody (1:500; cat. no. 2338052, Jackson ImmunoResearch Laboratories) for 1 h and DAPI for 5 min. Images were captured on a Zeiss fluorescent microscope in the green (MMP2) and blue (DAPI) channels. Images were also taken in the red channel to identify and avoid autofluorescent regions in the ovary, which are common in the aged ovary^43^. The mean fluorescent intensity from three MMP2-stained stromal regions from each mouse was quantified by Image J and averaged. Nonspecific background fluorescence was calculated in a similar manner using secondary-only stained serial sections from the same mouse. The nonspecific signal was subtracted from each sample.

### Flow cytometry

High-parameter spectral flow cytometry was performed to confirm age-related changes in the percentage and number of immune cells. C57BL/6 J (strain no. 000664) female mice (*n* = 30) were purchased from the Jackson Laboratory. To obtain sufficient cells for the gating strategy proposed, six ovaries from three mice were pooled (*n* = 5 per age group). Before euthanasia, vaginal cytology was performed to determine estrous cycle stage as previously described^92^. The mice were anesthetized with isoflurane and intravenously injected with 2 μg of FITC-anti-CD45 mAb (cat. no. 35–0454, Cytek) to label intravascular cells and distinguish them from tissue-resident extravascular leukocytes^116^. Five minutes after labeling, mice were euthanized and ovaries collected and pooled according to estrous cycle stage. Ovarian cells were isolated by enzymatic digestion in 3 ml of DMEM (cat. no. D6429, Sigma) containing 4 mg of collagenase (cat. no. C5138-100MG, Sigma-Aldrich). Samples were incubated at 37 °C for 40 min and gently pipetted 30 times every 10 min to encourage tissue dissociation^93^. Following dissociation, cells were passed through a 70-µm filter (cat. no. 130-098-462, Miltenyi Biotec) and washed with an additional 7 ml of DMEM. Cells were labeled with Zombie NIR (cat. no. 423106, BioLegend) according to the manufacturer’s instructions then washed in FACS Buffer (PBS + 5% newborn calf serum), incubated with a Fc blocking reagent (anti-mouse CD16/32, cat. no. 70-0161, Cytek), washed and stained with a surface-staining, fluorochrome-labeled mAb cocktail (Supplementary Data 1) for 30 min at 4 °C, in the presence of Brilliant Stain Buffer Plus (10 μl per sample; cat. no. 563795, Becton Dickinson) and CellBlox Blocking Buffer (5 μl per sample; cat. no. C001T02F01, Thermo Fisher Scientific). Cells were washed again in FACS buffer and intracellularly stained to detect transcription factor expression using the True-Nuclear Transcription Factor Buffer Set (cat. no. 424401, BioLegend) according to the manufacturer’s instructions. At the end of the procedure, stained cells were fixed in 2% paraformaldehyde for 5 min. Stained cells were acquired using a five-laser Cytek Aurora flow cytometer and analyzed using FlowJo 10.9 (Becton Dickinson). The gating strategy can be seen in Supplementary Fig. 4.

### Statistics and reproducibility

Differentially expressed genes between CLUs and by age were called in the Seurat package using the FindMarkers command with default parameters. Differentially expressed gene lists were imported into IPA software and filtered on false discovery rate (FDR) < 0.1 and log(fold change) > 0.25 for pathway analyses. Pathways with *P* < 0.05 were considered statistically significant, and activation *z*-scores are reported by heatmap or bar charts in the figures. More traditional statistical analyses were performed using GraphPad Prism software. The Shapiro–Wilk test was performed to confirm normal distribution of data. Strip plots are presented with individual points shown and means ± s.e.m. indicated. For comparison of means between the two ages, Student’s *t*-tests were used, applying one- and two-tailed tests where appropriate^117^. Corrections for multiple comparisons were made, where appropriate, using the Benjamini, Krieger and Yekutieli correction for multiple comparisons. Significant differences were defined at *P* < 0.05 or FDR < 0.05 (for multiple comparisons). No statistical methods were used to determine the number of biological replicates to use. Our sample size was selected based on sample sizes reported in previous similar publications^18,81^. Because the experimental groups used in this study were separated by age, it was not possible to randomize samples. No samples were removed from analysis. All criteria for data exclusion were pre-established. Cells with >400 unique molecular identifier counts, >200 genes or >25% of mitochondrial RNA counts were filtered. Also, cells expressing contradictory markers of known different cell types were removed as potential doublets. One of the cell CLUs was present in only one sample and presented oviduct cell markers rather than ovarian cell markers; this CLU was considered as oviduct tissue contamination and was removed from further analysis. Histological assessments were performed in a blinded manner. For single-cell library preparation, the sample order was randomized and the person preparing the samples blinded to sample grouping. For single-cell data analysis, blinding was not possible due to the statistical methods chosen for comparison by group. Each sample was given assigned group-identifying metadata. Identities have to be called during coding to assess differential expression between groups. It is not possible to perform the correct analysis without knowing from which group each identity is determined. All samples were treated equivalently during quality control steps.

### Reporting summary

Further information on research design is available in the Nature Portfolio Reporting Summary linked to this article.

### Supplementary information

Supplementary Figs. 1–8.

Reporting Summary

Supplementary Data 1 Supplementary data containing lists of genes and antibodies.

Supplementary Data 2 Statistical data for Supplementary Fig. 1.

Supplementary Data 3 Statistical data for Supplementary Fig. 4.

Supplementary Data 4 Statistical data for Supplementary Fig. 5.

Supplementary Data 5 Statistical data for Supplementary Fig. 6.

### Source data

Source Data Fig. 2 Statistical source data.

Source Data Fig. 3 Statistical source data.

Source Data Fig. 4 Statistical source data.

Source Data Fig. 6 Statistical source data.

## Data Availability

The datasets generated through this work are available in a publicly accessible repository. Raw and processed data files can be downloaded from NCBI Gene Expression Omnibus at accession no. GSE232309. An interactive Shiny-based web application is also available at https://omrf.shinyapps.io/OvarianAgingSCAtlas/. Sequencing data for the study of Morris et al.^26^ were retrieved from Open Science Framework and the Broad Institute Single Cell Portal under study no. SCP1914. Gene lists are available as Supplementary Data files. Source Data are provided with this paper.

## Code availability

The scRNA-seq data analysis was performed using scripts in R, which are available in GitHub: OvarianAgingAtlas (github.com/StoutLab/OvarianAgingAtlas).
