# Supplementary material for: A single-cell atlas of the aging mouse ovary
Source: Nat Aging. 2024 Jan 10;4(1):145–62. doi: 10.1038/s43587-023-00552-5 (PMC10798902; doi:10.1038/s43587-023-00552-5)
Supplement: Supplementary file 1 — Supplementary Figs. 1–8. [file 43587_2023_552_MOESM1_ESM.pdf]

nature aging

Resource

https://doi.org/10.1038/s43587-023-00552-5

A single-cell atlas of the aging mouse ovary

In the format provided by the authors and unedited

SUPPLEMENTARY INFORMATION FILE

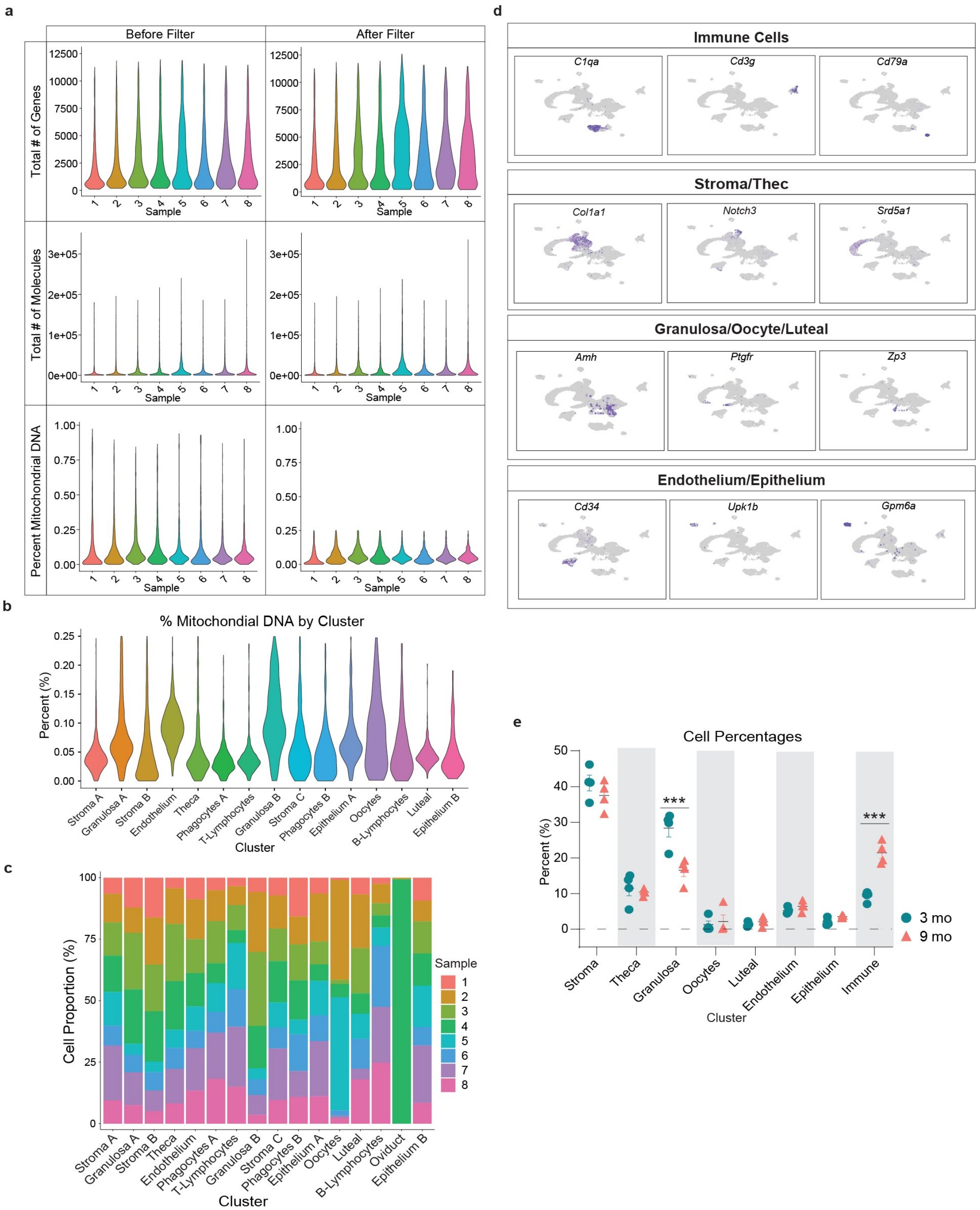

Suppl. Fig. 1. (a) Violin plots of the total number of genes and molecules and mitochondrial percentages within each sample before and after quality filtering. (b) Percent of mitochondrial RNA by CLU. (c) Percentage of cells in each CLU by sample, showing oviduct cells contamination in one sample, which was removed from further analyses. (d) Feature plots of specific marker genes of cell types used to identify the CLUs. (e) Cell percentages in each broad cell type by sample. scRNA-seq was performed in n=4 ovaries/age. Data are presented as mean ± SEM. *, **, *** represent statistical difference (FDR<0.05, 0.01 and 0.005, respectively) by multiple two-tailed t-test with Benjamini, Krieger, and Yekutieli correction for multiple comparisons Exact p-values are displayed in Source Data Suppl. Fig. 1.

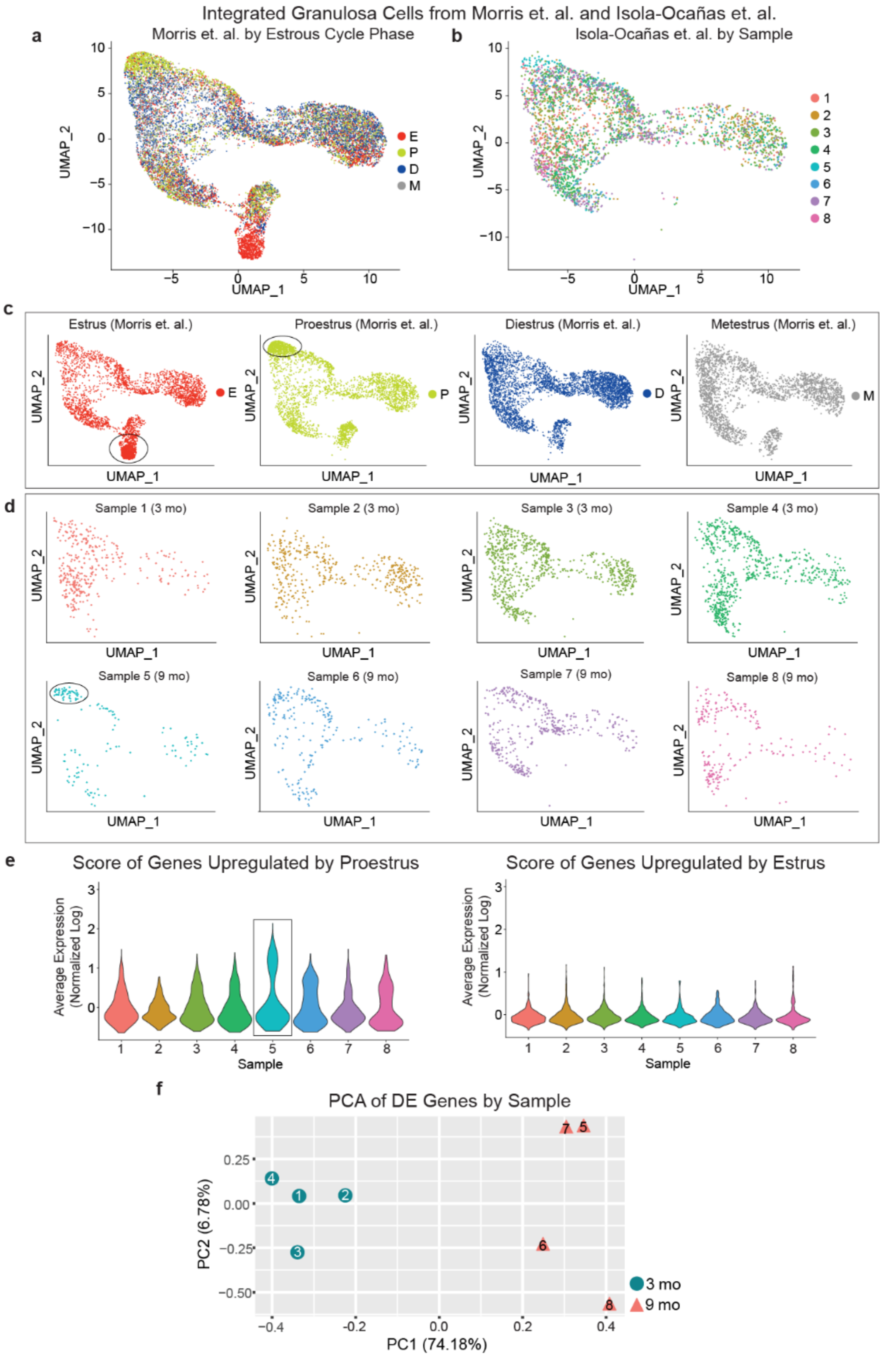

Suppl. Fig. 2. *In silico* estrous cycle staging by comparison to Morris et al., 2022. GC from the Morris et al. study were co-clustered with GC from the present study to infer estrous cycle stage. (a) UMAP plot featuring GC across different estrous cycle stages from Morris et al. (b) UMAP plot featuring GC from all samples of the present study integrated with Morris et al. dataset. (c) UMAP plots from Morris et al. featuring each phase of estrous cycle, noting distinct CLUs only observed during the estrus and proestrus phases. (d) UMAP plot featuring GC from each samples of the present study, suggesting Sample 5 is in proestrus and the remaining samples are in either metestrus or diestrus. (e) Module score of genes reported by Morris et al. as upregulated in GC in both estrus and proestrus in each of our samples, suggesting Sample 5 is in proestrus and the remaining samples are in either metestrus or diestrus. (f) PCA of gene expression by sample, showing clear separation of samples by age. scRNA-seq was performed in n=4 ovaries/age.

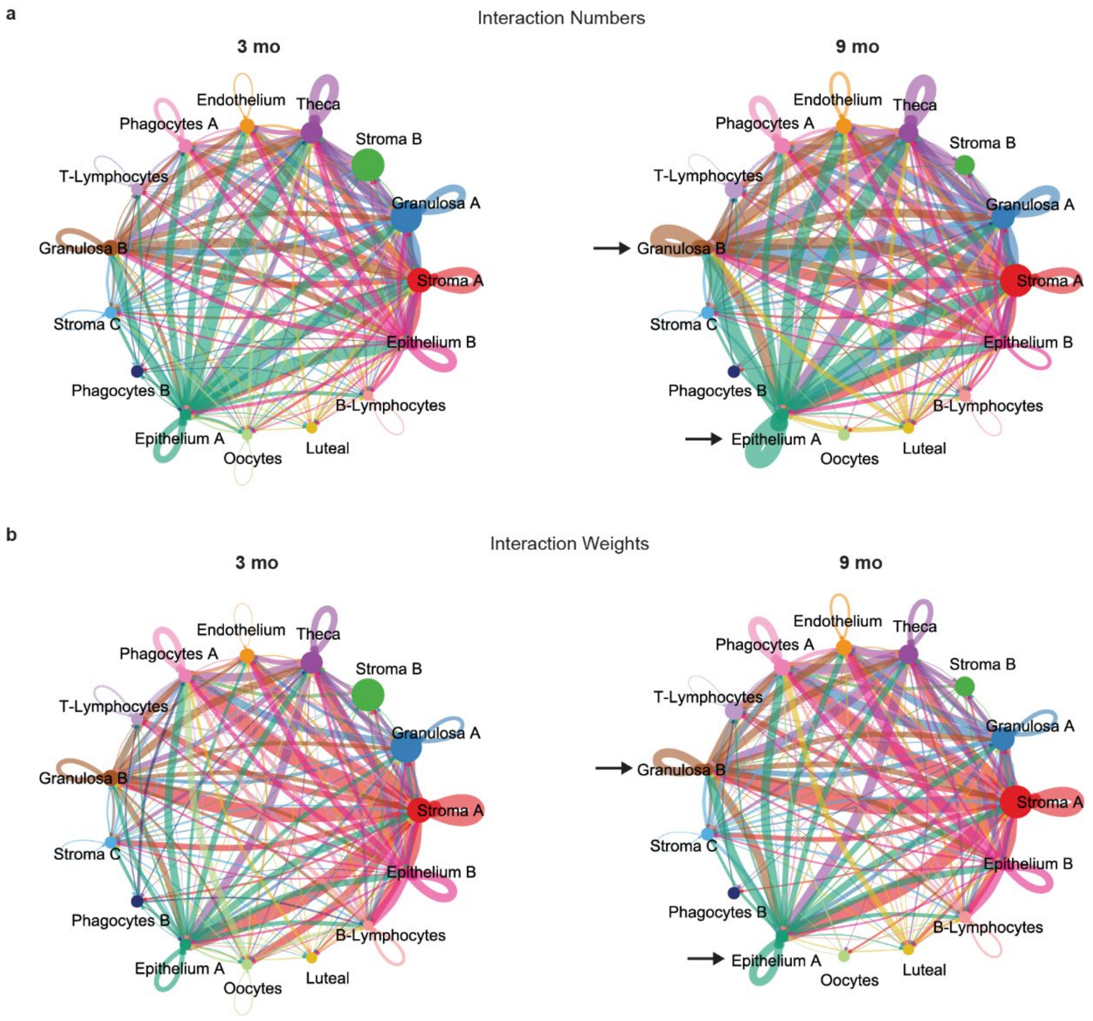

Suppl. Fig. 3. Cellular communication networks in the aging murine ovary. (a) Number of cellular interactions in the 3-mo and 9-mo ovarian using the CellChat signaling package. (b) Interaction weights in the 3-mo and 9-mo ovarian using the CellChat signaling package. scRNA-seq was performed in n=4 ovaries/age.

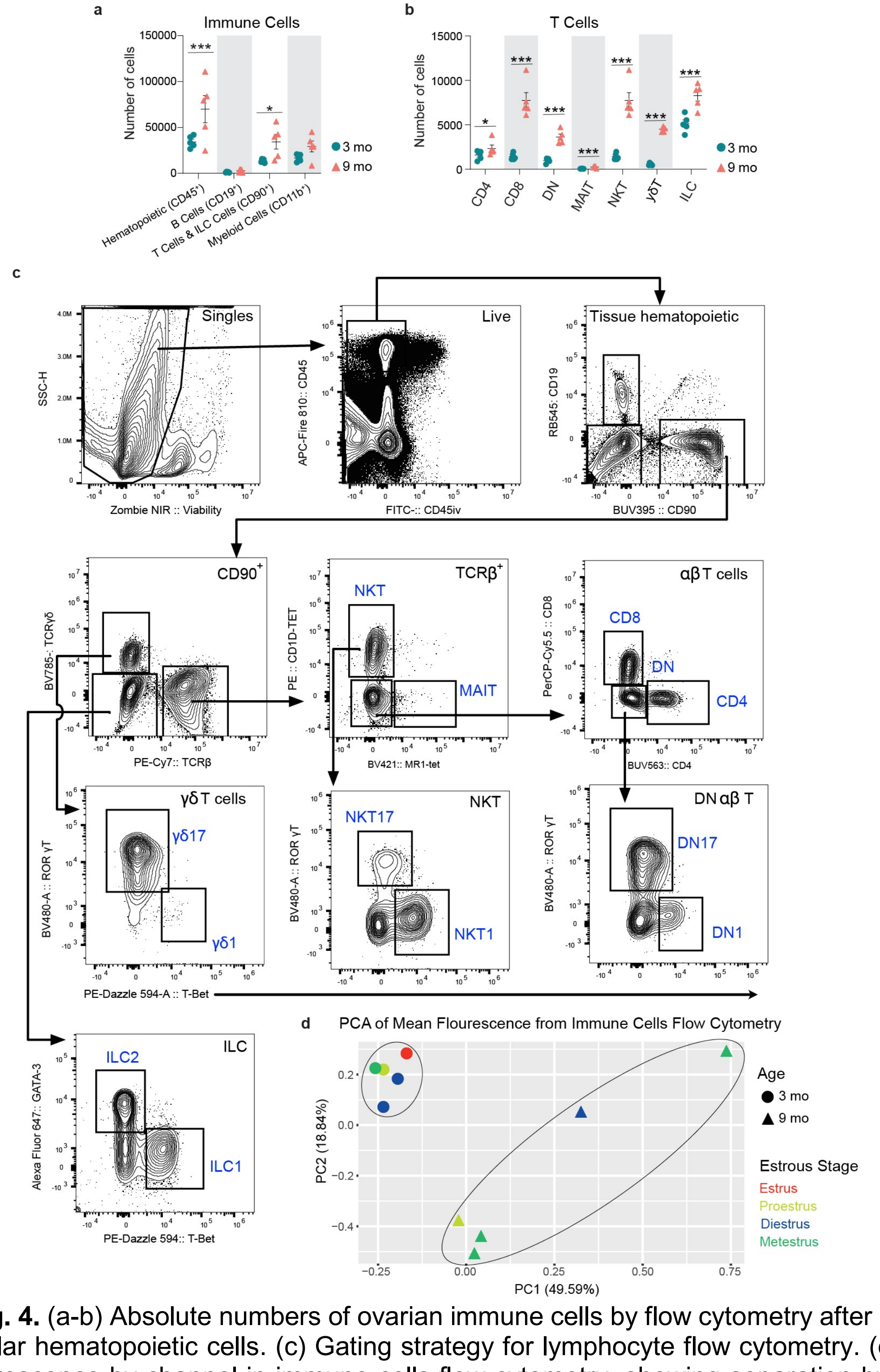

Suppl. Fig. 4. (a-b) Absolute numbers of ovarian immune cells by flow cytometry after gating out intravascular hematopoietic cells. (c) Gating strategy for lymphocyte flow cytometry. (d) PCA of mean fluorescence by channel in immune cells flow cytometry, showing separation by age and not estrous cycle stage. For flow cytometry, 6 ovaries from mice in the same phase of estrous cycle were pooled together to comprise each sample (n=5/age). Data are presented as mean ± SEM. *, **, *** represent statistical difference (FDR<0.05, 0.01 and 0.005, respectively) by multiple two-tailed t-test with Benjamini, Krieger, and Yekutieli correction for multiple comparisons. Exact p-values are displayed in Source Data Suppl. Fig. 4.

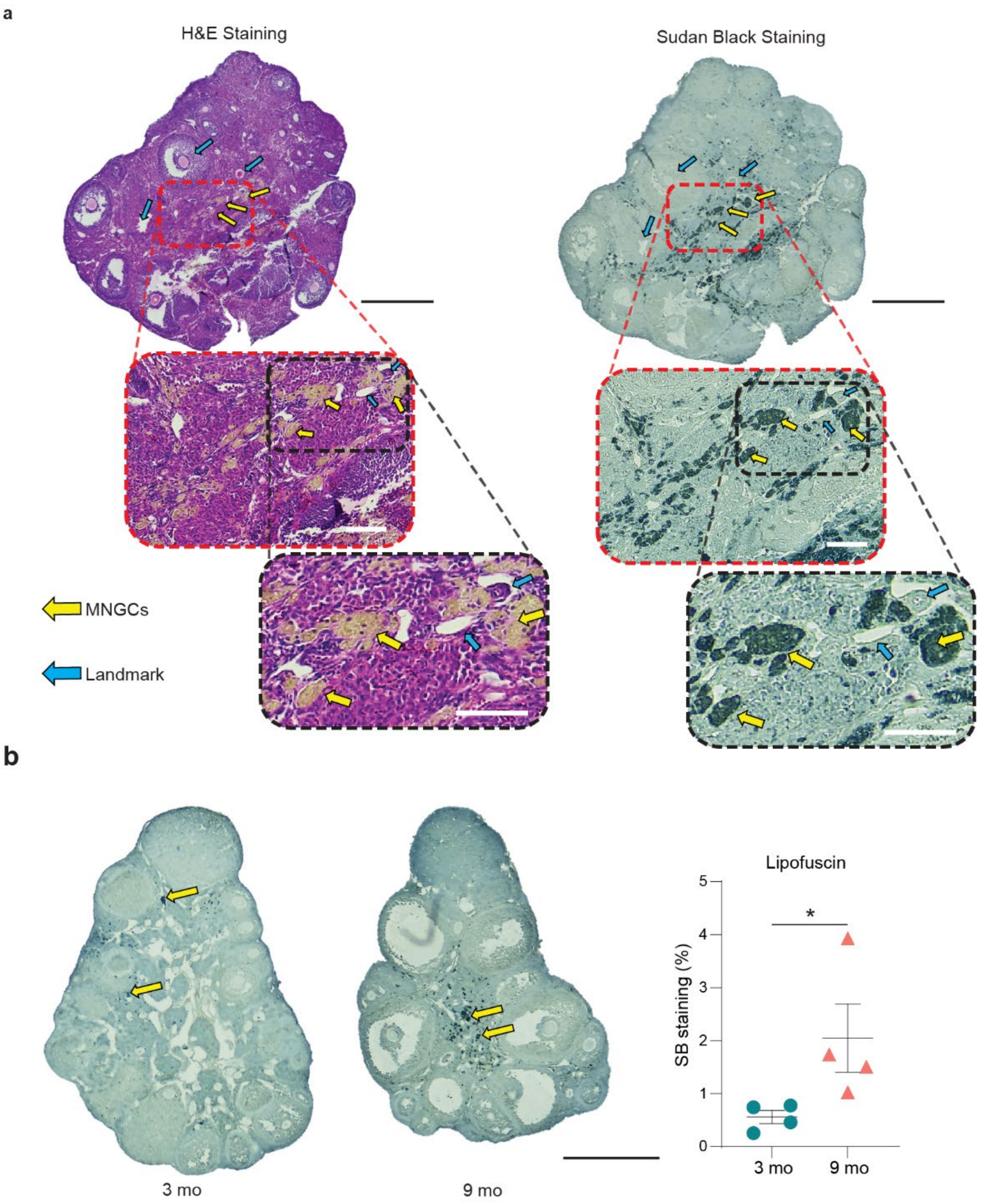

Suppl. Fig. 5. Assessment of lipofuscin accumulation in the aged ovary. (a) H&E and Sudan black staining of 9-month-old ovarian sections showing MNGC accumulation compared to 3-month-old ovaries. (b) Sudan black staining of lipofuscin in 3- and 9-month-old ovaries. Data are presented as mean ± SEM. * represents statistical difference ($p<0.05$) by one-tailed t-test. scRNA-seq was performed in n=4 ovaries/age. Black scale bars represent 500μm and white scale bars represent 100μm. Exact p-values are displayed in Source Data Suppl. Fig. 5.

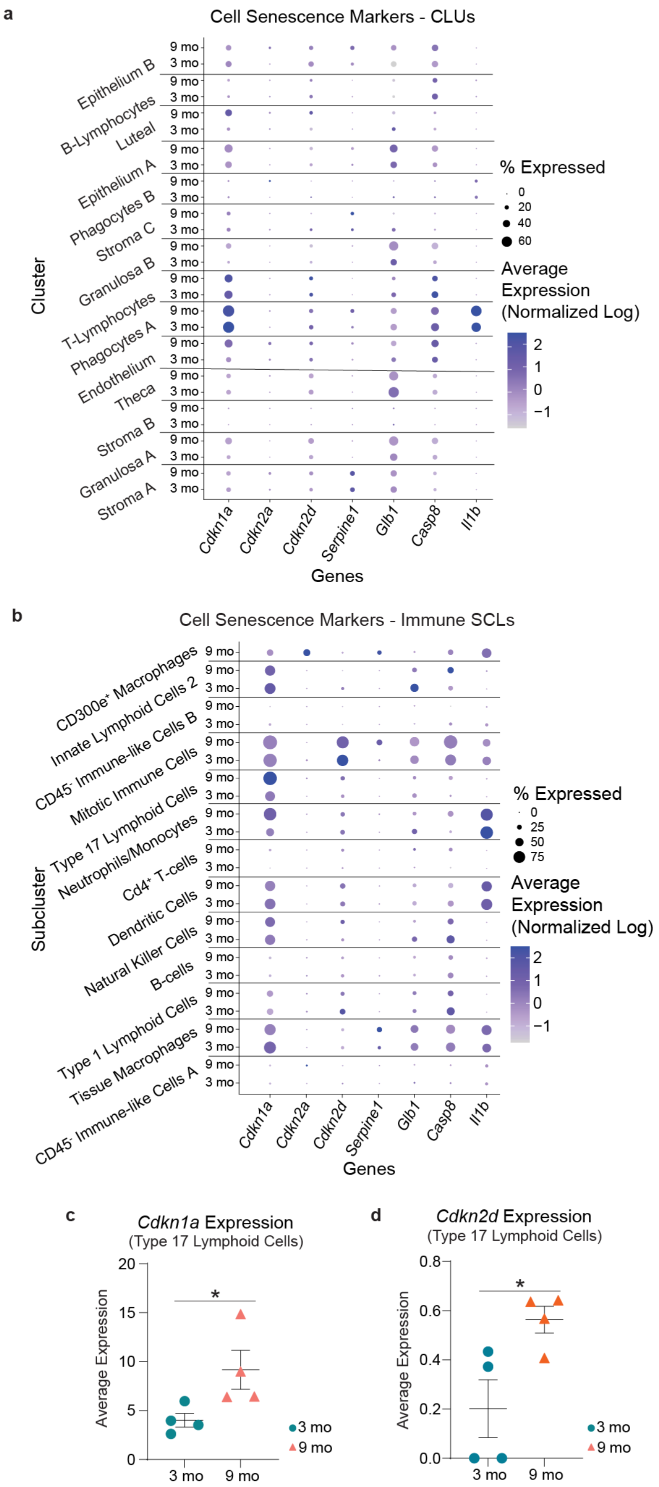

Suppl. Fig. 6. Expression of cellular senescence markers is unchanged with age. (a) Dot plot of expression of cellular senescence markers in initial CLUs. (b) Dot plot of expression of cell senescence markers in immune SCLs. (c) *Cdnk1a* expression in Type 17 lymphoid cells. (d) *Cdnk2a* expression in Type 17 lymphoid cells. scRNA-seq was performed in n=4 ovaries/age. Data are presented as mean ± SEM. *, **, *** represent statistical difference (p<0.05, 0.01 and 0.005, respectively) by one-tailed t-test. Exact p-values are displayed in Source Data Suppl. Fig. 6.

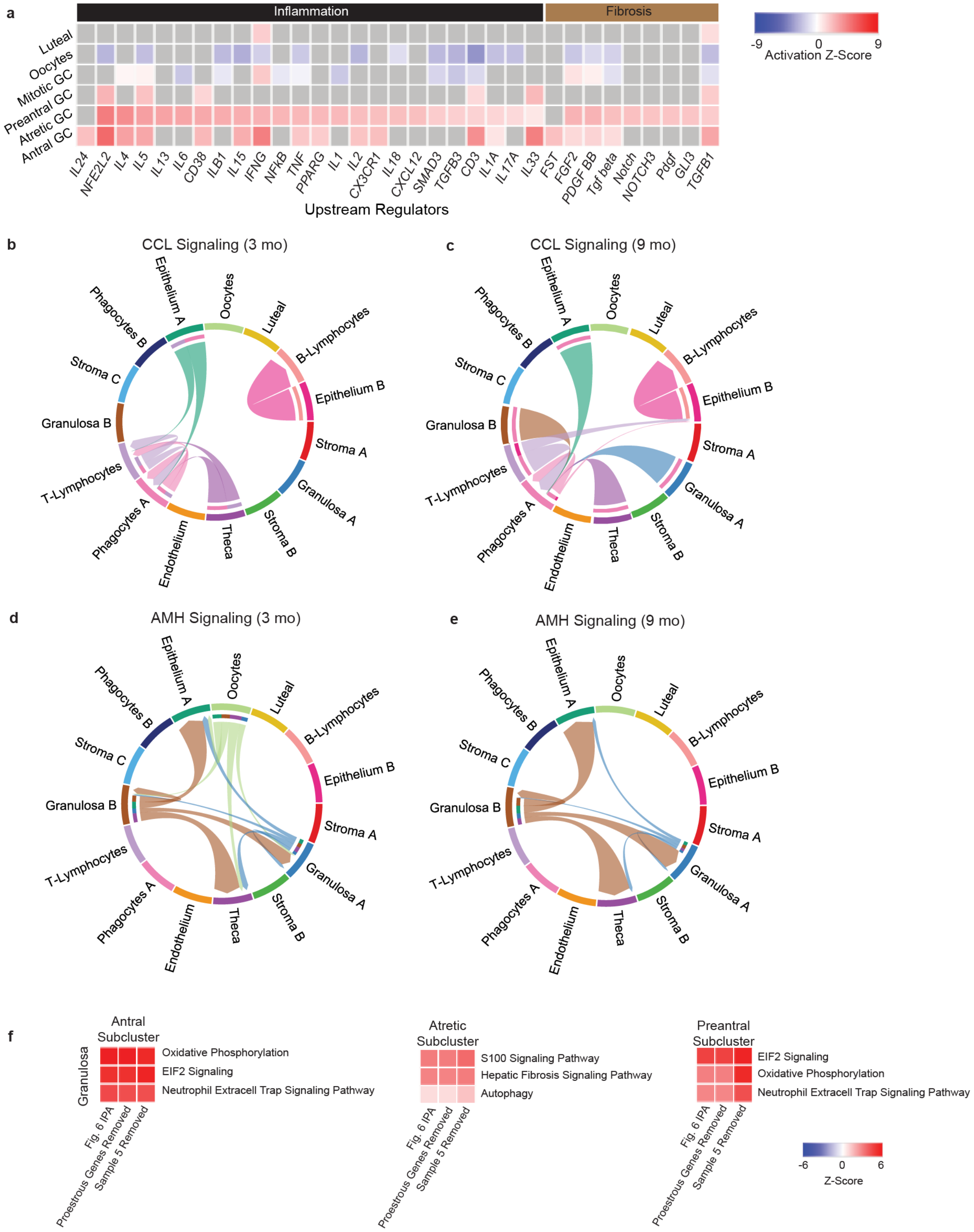

Suppl. Fig. 7. Cell signaling in granulosa cells, oocytes and luteal cells. (A) Z-scores indicating activation or inhibition of upstream regulators with aging by IPA analysis in granulosa/oocytes/luteal SCLs. (B-C) CellChat chord diagrams of the CCL signaling pathway interactions in the 3- and 9-month-old ovarian CLUs. (D-E) CellChat chord diagrams of the AMH signaling pathway interactions in the 3- and 9-month-old ovarian CLUs. (F) Heatmaps of three of the most changed IPA pathways from Fig. 6 in granulosa SCLs with and without the removal of proestrus upregulated genes or sample 5, showing little to no changes. scRNA-seq was performed in n=4 ovaries/age.
