# Supplementary material for: A single-cell atlas of the aging mouse ovary
Source: Nat Aging. 2024 Jan 10;4(1):145–62. doi: 10.1038/s43587-023-00552-5 (PMC10798902; doi:10.1038/s43587-023-00552-5)
Supplement: Supplementary file 2 — Reporting Summary [file 43587_2023_552_MOESM2_ESM.pdf]

nature portfolio

Corresponding author(s): Michael Stout

Last updated by author(s): 10/31/2023

Reporting Summary

Nature Portfolio wishes to improve the reproducibility of the work that we publish. This form provides structure for consistency and transparency in reporting. For further information on Nature Portfolio policies, see our Editorial Policies and the Editorial Policy Checklist.

Statistics

For all statistical analyses, confirm that the following items are present in the figure legend, table legend, main text, or Methods section.

n/a	Confirmed	
☐	☒	The exact sample size (*n*) for each experimental group/condition, given as a discrete number and unit of measurement
☐	☒	A statement on whether measurements were taken from distinct samples or whether the same sample was measured repeatedly
☐	☒	The statistical test(s) used AND whether they are one- or two-sided<br>*Only common tests should be described solely by name; describe more complex techniques in the Methods section.*
☐	☒	A description of all covariates tested
☐	☒	A description of any assumptions or corrections, such as tests of normality and adjustment for multiple comparisons
☐	☒	A full description of the statistical parameters including central tendency (e.g. means) or other basic estimates (e.g. regression coefficient) AND variation (e.g. standard deviation) or associated estimates of uncertainty (e.g. confidence intervals)
☐	☒	For null hypothesis testing, the test statistic (e.g. *F*, *t*, *r*) with confidence intervals, effect sizes, degrees of freedom and *P* value noted<br>*Give P values as exact values whenever suitable.*
☒	☐	For Bayesian analysis, information on the choice of priors and Markov chain Monte Carlo settings
☒	☐	For hierarchical and complex designs, identification of the appropriate level for tests and full reporting of outcomes
☒	☐	Estimates of effect sizes (e.g. Cohen's *d*, Pearson's *r*), indicating how they were calculated

Our web collection on statistics for biologists contains articles on many of the points above.

Software and code

Policy information about availability of computer code

Data collection	NovaSeq 6000 System used to generate sequencing data.
Data analysis	Generated sequencing data was analyzed using QIAGEN Ingenuity Pathway Analysis (https://digitalinsights.qiagen.com/products-overview/discovery-insights-portfolio/analysis-and-visualization/qiagen-ipa/), RStudio (http://www.rstudio.com/, version 4.2.2), SoupX (https://github.com/constantAmateur/SoupX), CellRanger (https://support.10xgenomics.com/single-cell-gene-expression/software/overview/welcome, version 3.1), Seurat (https://github.com/satijalab/seurat, version 4.3.0), SeuratWrappers (https://github.com/satijalab/seurat-wrappers, version 0.3.1), DoubletFinder (https://github.com/chris-mcginnis-ucsf/DoubletFinder, version 2.0.3), scCustomize(https://github.com/samuel-marsh/scCustomize, version 1.1.1), ShinyCell (https://github.com/SGDDNB/ShinyCell, version 2.1.0), CellChat (https://github.com/sqjin/CellChat, version 1.6.1). In-house scripts can be accessed at https://github.com/StoutLab/OvarianAgingAtlas.

For manuscripts utilizing custom algorithms or software that are central to the research but not yet described in published literature, software must be made available to editors and reviewers. We strongly encourage code deposition in a community repository (e.g. GitHub). See the Nature Portfolio guidelines for submitting code & software for further information.

Data

Policy information about availability of data

All manuscripts must include a data availability statement. This statement should provide the following information, where applicable:

- Accession codes, unique identifiers, or web links for publicly available datasets
- A description of any restrictions on data availability
- For clinical datasets or third party data, please ensure that the statement adheres to our policy

The datasets generated through this work are available in a publicly accessible repository. Raw and processed data files can be downloaded from NCBI Gene Expression Omnibus at accession number GSE232309. An interactive Shiny-based web application is also available at https://omrf.shinyapps.io/OvarianAgingSCAtlas/. Sequencing data for the Morris et al. (2022) study were retrieved from Open Science Framework and the Broad Institute Single Cell Portal under study number SCP1914. Gene lists are available are supplementary data files and data underlying dot plots is available as source data files.

Research involving human participants, their data, or biological material

Policy information about studies with human participants or human data. See also policy information about sex, gender (identity/presentation), and sexual orientation and race, ethnicity and racism.

Reporting on sex and gender	NA
Reporting on race, ethnicity, or other socially relevant groupings	NA
Population characteristics	NA
Recruitment	NA
Ethics oversight	NA

Note that full information on the approval of the study protocol must also be provided in the manuscript.

Field-specific reporting

Please select the one below that is the best fit for your research. If you are not sure, read the appropriate sections before making your selection.

☒ Life sciences ☐ Behavioural & social sciences ☐ Ecological, evolutionary & environmental sciences

For a reference copy of the document with all sections, see nature.com/documents/nr-reporting-summary-flat.pdf

Life sciences study design

All studies must disclose on these points even when the disclosure is negative.

Sample size	For scRNA-Seq, n=4 biological replicates/group were sequenced with ~2000 cells/sample. No statistical methods were used to determine the number of biological replicates to use. Our sample size was selected based on sample sizes reported in previous similar publications (PMID: 32004457, 33351795, 33378681, 37096871). Additionally, we used SCOPIT Power calculations to determine that ~12,000 cells were needed to observe >100 cells per cluster, assuming that the rarest population was 1% of the total cells. Thus, we targeted 16,000 cells to ensure enough cells were available to make conclusions about rare populations.
Data exclusions	No samples were removed from analysis. All criteria for data exclusion were pre-established. Cells with less than 400 UMI counts, less than 200 genes, or greater than 25% of mitochondrial RNA counts were filtered. Also, cells expressing contradictory markers of known different cell types were removed as potential doublets. One of the cell clusters was present in only one sample and presented oviduct cell markers rather than ovarian cell markers. This cluster was considered oviduct tissue contamination and removed from further analysis.
Replication	Four biological replicates were included for each group. All biological replicates were successful and included in analysis. Single-cell findings were verified by orthogonal methods where possible, including flow cytometry and histological assessments. Findings were compared and integrated with previously published datasets.
Randomization	Since the experimental groups used in this study were separated by age, it was not possible to randomize samples. Mice were purchased from the Jackson Laboratory at the intended ages.
Blinding	Histological assessments were performed in a blinded manner. For single-cell library preparation, the sample order was randomized and the person preparing the samples were blinded to the sample grouping. For single-cell data analysis, blinding was not possible due to the statistical methods chosen to compare by group. Each sample was given assigned group-identifying meta-data. The identities have to be called during coding in order to assess differential expression between groups. It is not possible to perform the correct analysis without knowing from which group each identity is from. All samples were treated equivalently during quality control steps.

Reporting for specific materials, systems and methods

We require information from authors about some types of materials, experimental systems and methods used in many studies. Here, indicate whether each material, system or method listed is relevant to your study. If you are not sure if a list item applies to your research, read the appropriate section before selecting a response.

Materials & experimental systems

n/a	Involved in the study
☐	☒ Antibodies
☒	☐ Eukaryotic cell lines
☒	☐ Palaeontology and archaeology
☐	☒ Animals and other organisms
☒	☐ Clinical data
☒	☐ Dual use research of concern
☒	☐ Plants

Methods

n/a	Involved in the study
☒	☐ ChIP-seq
☐	☒ Flow cytometry
☒	☐ MRI-based neuroimaging

Antibodies

Antibodies used

Flow cytometry
CD45.2 FITC 104 1 mg/ml Cytek Biosciences (Cat. No. 35-0454)
CD90 BUV395 53-2.1 1/100 BD Biosciences (Cat. No. 740205)
CD4 BUV563 GK1.5 1/400 BD Biosciences (Cat. No. 612923)
CD69 BUV737 H1.2F3 1/200 BD Biosciences (Cat. No. 612793)
MRI-5-OP-RU-TET BV421 - 1/400 NIH Tetramer Facilities
Ly-6G BV510 1A8 1/100 Biolegend (Cat. No. 127633)
CD44 BV650 IM7 1/100 Biolegend (Cat. No. 103049)
CD103 BV711 M290 1/100 BD Biosciences (Cat. No. 564320)
TCRgd BV786 GL3 1/100 BD Biosciences (Cat. No. 740995)
CD19 RB545 103 1/100 BD Biosciences (Cat. No. 569727)
CDllb NFB610-30S MI/70 1/100 Thermo Fisher Scientific (Invitrogen) (Cat. No. M015T02B05)
CD8a PerCP-Cy5.5 53-6. 7 1/100 Biolegend (Cat. No. 100734)
CDld-PBS-57-TET PE - 1/800 NIH Tetramer Core Facility
B220 PE-F700 RA3-6B2 1/100 Biolegend (Cat. No. 103280)
TCRb PE-Cy7 H57-597 1/100 Biolegend (Cat. No. 109222)
CD49b APC DX5 1/100 Biolegend (Cat. No. 108910)
MHC 11 AlexaFluor700 M5/114.15.2 1/200 Biolegend (Cat. No. 107622)
CD45 APC Fire 810 30-Fll 1/100 Biolegend (Cat. No. 103174)
CellBlox Blocking Buffer 5 ul/test Thermo Fisher Scientific (lnvitrogen) (Cat. No. B001T06F01)
Brilliant Stain Buffer Plus 10 uL/test BD Biosciences (Cat. No. 566385)
RORgT BV480 Q31-378 1/100 BD Biosciences (Cat. No. 567176)
GATA-3 AF647 L50-823 1/100 BD Biosciences (Cat. No. 560068)
T-bet PE/Dazzle 594 4B10 1/200 Biolegend (Cat. No. 644828)

lmmunofluorescence
MMP-2 (D2O4T) Rabbit mAb 1/100 Cell Signaling (Cat. No. 87809)
IgG Alexa Fluor 488 antibody 1/500 Jackson ImmunoResearch Laboratories (Cat. No. 2338052)

Validation

The CD1d and MR1 tetramers are not commercially available. They were obtained from the NIH Tetramer Core Facility (https://tetramer.yerkes.emory.edu/) and validated on normal mouse tissues using negative control tetramers supplied by the same facility. All other antibodies used were commercially available and previously validated in mice for the application used, as stated in the manufacturers websites: https://www.bdbiosciences.com, https://www.biolegend.com/en-gb, https://www.thermofisher.com/us/en/home/brands/invitrogen, and https://www.cellsignal.com. Antibodies were also validated in-house uunder the staining conditions used.

Animals and other research organisms

Policy information about studies involving animals; ARRIVE guidelines recommended for reporting animal research, and Sex and Gender in Research

Laboratory animals

3- and 9-month-old C57Bl/6J female mice. Mice were kept at 22 ± 0.5°C on a 12:12-hour light-dark cycle and had ad libitum access to food and water.

Wild animals

No wild animals were used in the study.

Reporting on sex

Since this is an ovarian aging study we only evaluated female mice.

Field-collected samples	No field-collected samples were used in the study.
Ethics oversight	This study was approved by the Oklahoma Medical Research Foundation IACUC and performed in accordance with institutional and national guidelines.

Note that full information on the approval of the study protocol must also be provided in the manuscript.

Flow Cytometry

Plots

Confirm that:

☒ The axis labels state the marker and fluorochrome used (e.g. CD4-FITC).

☒ The axis scales are clearly visible. Include numbers along axes only for bottom left plot of group (a 'group' is an analysis of identical markers).

☒ All plots are contour plots with outliers or pseudocolor plots.

☒ A numerical value for number of cells or percentage (with statistics) is provided.

Methodology

Sample preparation	To obtain enough cells for the gating strategy proposed, six ovaries from three mice were pooled (n=5/age). Ovarian cells were isolated by enzymatic digestion in 3 mL of Dulbecco's Modified Eagle's Medium (DMEM) (cat# D6429, Sigma) containing 4 mg collagenase (cat# C5138-100MG, Sigma-Aldrich, St. Louis, MO). Samples were incubated at 37°C for 40 minutes and gently pipetted 30 times every 10 minutes to encourage tissue dissociation93. Following dissociation, cells were passed through a 70 µm filter (cat# 130-098-462, Miltenyi Biotec) and washed with an additional 7 mL of DMEM. Cells were labeled with Zombie NIR (cat# 423106, Biolegend, San Diego, CA, USA) according to the manufacturer's instructions, then washed in FACS Buffer (PBS + 5% Newborn Calf Serum), incubated with a Fc blocking reagent (anti-mouse CD16/32; cat# 70-0161, Cytek), washed and stained with a surface staining fluorochrome-labeled mAb cocktail (Suppl. File 7) for 30 min at 4º C, in the presence of Brilliant Stain Buffer Plus (10 µl/sample) (cat# 563795, Becton Dickinson, Mountain View, Ca, USA) and CellBlox Blocking Buffer (5 µl/sample) (cat# C001T02F01, Thermo Fisher Scientific). Cells were washed again in FACS buffer and intracellularly stained to detect transcription factor expression using the True-Nuclear Transcription Factor Buffer Set (cat# 424401, Biolegend) according to the manufacturer's instructions. At the end of the procedure, stained cells were fixed in 2% paraformaldehyde for 5 min.
Instrument	5-laser Cytek Aurora
Software	FlowJo 10.9 (Becton Dickinson)
Cell population abundance	No sorting was performed. To assure sufficient number of cells in the distinct cell populations after gating, 6 ovaries were pooled for each sample.
Gating strategy	For the analysis of the flow cytoemtry data we first excluded doublets using a FSH-A/FSH-C histogram. Dead cells were then excluded based on Zombie NIR staining. We then gated on tissue hematopoietic cells as CD45+ i.v. CD45-. The resulting tissue hematopoietic were then sequentially gated as shown in Supplementary Figure 4.

☒ Tick this box to confirm that a figure exemplifying the gating strategy is provided in the Supplementary Information.
